# Association between polygenic risk for Major Depression and brain structure in a mega-analysis of 50,975 participants across 11 studies

**DOI:** 10.1038/s41380-025-03136-4

**Published:** 2025-08-19

**Authors:** Xueyi Shen, Yara J. Toenders, Laura K. M. Han, Antoine Weihs, Nina Alexander, Till F. M. Andlauer, Katharina Brosch, Andreas J. Forstner, Dominik Grotegerd, Tim Hahn, Marco Hermesdorf, Norbert Hosten, Hamidreza Jamalabadi, Susanne Meinert, Yuri Milaneschi, Philipp G. Sämann, Frederike Stein, Aleks Stolicyn, Lea Teutenberg, Gladi Thng, Mark J. Adams, Florian Thomas-Odenthal, Paula Usemann, Uwe Völker, Katharina Wittfeld, Marisol Herrera-Rivero, Yunxuan Jiang, Chao Tian, Nynke A. Groenewold, Sheri-Michelle Koopowitz, Lachlan T. Strike, Udo Dannlowski, Andreas Jansen, Tilo Kircher, Igor Nenadić, Kang Sim, Benjamin Straube, Henry Völzke, Dan J. Stein, Sarah E. Medland, Klaus Berger, Hans J. Grabe, Axel Krug, Katie L. McMahon, Greig de Zubicaray, Elena Pozzi, Dick J. Veltman, Sophia I. Thomopoulos, Neda Jahanshad, Paul M. Thompson, Lianne Schmaal, Andrew M. McIntosh, Heather C. Whalley

**Affiliations:** 1 Division of Psychiatry, University of Edinburgh, Edinburgh, UK; 2 Erasmus School of Social and Behavioral Sciences, Erasmus University Rotterdam, Rotterdam, The Netherlands; 3 Centre for Youth Mental Health, The University of Melbourne, Parkville, VIC Australia; 4 Orygen, Parkville, VIC Australia; 5 Department of Psychiatry, Amsterdam UMC, Vrije Universiteit Amsterdam, Amsterdam, The Netherlands; 6 Department of Psychiatry and Psychotherapy, University Medicine Greifswald, Greifswald, Germany; 7 German Center for Neurodegenerative Diseases (DZNE), Site Rostock/Greifswald, Germany; 8 Department of Psychiatry and Psychotherapy, University of Marburg, Marburg, Germany; 9 Department of Neurology, Klinikum rechts der Isar, School of Medicine, Technical University of Munich, Munich, Germany; 10 Institute of Human Genetics, University of Bonn, School of Medicine & University Hospital Bonn, Bonn, Germany; 11 Institute of Neuroscience and Medicine (INM-1), Research Centre Jülich, Jülich, Germany; 12 Centre for Human Genetics, Philipps-University Marburg, Marburg, Germany; 13 Institute for Translational Psychiatry, University of Münster, Münster, Germany; 14 Institute of Epidemiology and Social Medicine, University of Münster, Münster, Germany; 15 Institute of Diagnostic Radiology and Neuroradiology, University Medicine Greifswald, Greifswald, Germany; 16 Institute for Translational Neuroscience, University of Münster, Münster, Germany; 17 Amsterdam Public Health, Mental Health program, Amsterdam, The Netherlands; 18 Amsterdam Neuroscience, Complex Trait Genetics, Amsterdam, The Netherlands; 19 Max Planck Institute of Psychiatry, Munich, Germany; 20 Interfaculty Institute of Genetics and Functional Genomics, University Medicine Greifswald, Greifswald, Germany; 21 23andMe, San Francisco, CA USA; 22 Department of Psychiatry and Neuroscience Institute, University of Cape Town, Cape Town, South Africa; 23 Psychiatric Genetics, QIMR Berghofer Medical Research Institute, Brisbane, QLD Australia; 24 West Region, Institute of Mental Health, Singapore, Singapore; 25 Yong Loo Lin School of Medicine, National University of Singapore, Singapore, Singapore; 26 Lee Kong Chian School of Medicine, Nanyang Technological University, Singapore, Singapore; 27 Institute for Community Medicine, University Medicine Greifswald, Greifswald, Germany; 28 SAMRC Research Unit on Risk and Resilience in Mental Disorders, University of Cape Town, Cape Town, South Africa; 29 Department of Psychiatry and Psychotherapy, University Hospital Bonn, Bonn, Germany; 30 School of Clinical Sciences, Queensland University of Technology, Brisbane, QLD Australia; 31 School of Psychology and Counselling, Queensland University of Technology, Brisbane, QLD Australia; 32 Department of Psychiatry, Amsterdam University Medical Center, Amsterdam, The Netherlands; 33 Imaging Genetics Center, Mark and Mary Stevens Neuroimaging and Informatics Institute, Keck School of Medicine, University of Southern California, Los Angeles, CA USA

**Keywords:** Depression, Predictive markers, Genetics, Neuroscience

## Abstract

Major Depression (MD) is a prevalent, disabling and life-limiting condition. The neurobiological associations of genetic risk for MD remain under-explored in large samples, with no comprehensive mega-analysis conducted to date. Our study analysed data from 11 separate studies, encompassing 50,975 participants from the ENIGMA Major Depressive Disorder Working Group. We developed highly consistent genetic and neuroimaging protocols and applied these throughout all participating studies, together with rigorous genetic methods to remove overlap between the polygenic risk scores (PRS) training and testing samples. Elevated PRS for MD correlated with lower intracranial volume and lower global measure of cortical surface area (β_ICV_ = −0.017, p_ICV_ = 1.97 × 10^−6^; β_Surf_ = −0.013, p_Surf_ = 4.5 × 10^−4^; pFDR < 3.62 × 10^−4^). The most significant cortical association was observed in the surface area of the frontal lobe (β = −0.011, *p* = 2.85 × 10^−6^, pFDR = 1.42 × 10^−5^), particularly in the left medial orbito-frontal gyrus (β = −0.021, *p* = 9.48 × 10^−8^, pFDR = 1.25 × 10^−5^). In subcortical regions, lower volumes of the thalamus, hippocampus, and pallidum correlated with higher PRS of MD (β ranged from −0.011 to −0.015, p ranged from 0.002–1.73 × 10^−5^, pFDR < 0.006). In a subsample of young individuals only (<25 years old, *N* = 5570), although there were no FDR-significant findings, directions of effects were highly consistent between the analyses of cortical surface areas in youth and the full sample (71.2% in the same direction, exact binomial test p-value = 7.56 × 10^−4^). Subsequent Mendelian randomisation analysis revealed potentially causal effects of smaller left hippocampal volume on higher liability for MD (Inverse variance weighted analysis β = −0.064, *p* = 8.04 × 10^−3^, pFDR = 0.04). Our findings represent an example of how extensive international collaborations can significantly advance our neurogenetic understanding of MD and give insights to avenues for early interventions in those at high risk for developing MD.

## Introduction

Major Depression (MD) is a leading contributor to global health burden, costing $0.7 trillion per year globally [1]. MD is a heritable condition with a twin-based heritability of 37% [2] and a SNP-estimated heritability of 6% [3]. Recent large genome-wide association studies (GWAS) of MD, a broader trait that is highly genetically correlated with clinically defined Major Depressive Disorder, have enabled the creation of polygenic risk scores (PRS) that can objectively estimate liability for MD [3]. Further, PRS can be used to facilitate association analyses in a wide range of health outcomes and biomarkers, and thereby help identify underlying disease mechanisms [4].

Previous large-scale studies have revealed cortical and subcortical structural brain abnormalities associated with MD diagnosis [5, 6], including case-control differences in hippocampal volumes [7]. Another meta-analysis revealed that MD was associated with lower cortical thickness in orbitofrontal cortex, cingulate, insula, and the temporal lobes [5]. In GWAS of MD, strong evidence has emerged for enrichment of brain-expressed genes and those involved in synaptic pathways [3]. These findings suggest that the structural brain differences and MD may have shared genetic architecture [8], although this has not been explored on a large scale.

Although neuroanatomical associations with genetic risk of MD have been implicated in previous studies, there have been limited large-scale studies examining the association between genetic risk and neuroimaging measures in MD, particularly across multiple cohorts [9]. Previous single-cohort studies often lacked sufficient statistical power to detect reliable associations with small effect sizes [10], and therefore tended to produce inconclusive findings in varying brain regions and inconsistent directions of effect [11]. Differences in pre-processing of imaging data between studies also introduce heterogeneity and may lead to lower reproducibility [12], especially in small brain regions [13]. Additionally, there has been no individual-level mega-analysis looking at the genetic risk of MD and structural brain variations [11]. The lack of large-scale neuroimaging and genetic datasets and the difficulty in harmonising analysis protocols have been major obstacles contributing to the lack of well-powered studies of this type [14, 15], and explains why this approach has rarely been implemented. Finally, previous studies mainly focused on mid- to late-life adults, with limited analyses of younger individuals [16]. This leads to difficulty in generalising findings across the lifespan [16]. Thus, a large-scale, multi-cohort study with representation across a wide age range is a crucial step to deepen our understanding of the neurogenetic basis of MD, and to potentially allow further identification of causal neural biomarkers.

Here, we conducted a unique, individual-level mega-analysis (*N* = 50,975) of participants from 11 studies to examine associations between PRS for MD, cortical thickness and surface area, and subcortical volumes. Standardised imaging and genetic protocols were used to harmonise genetic and neuroimaging data across cohorts. Our study sample included participants from a wide age range (mean age per cohort varied between 9–65 years old), with follow-up analyses that particularly focused on young individuals aged ≤25 years (*N* = 5570). For structural brain measures associated with PRS for MD, a follow-up bidirectional Mendelian randomisation analysis was conducted to test if there was any potential causal relationship between structural variation and liability for MD.

## Methods

### Participants

A total of 50,975 participants with European ancestry from nine cohorts of the ENIGMA Major Depressive Disorder working group [5, 7], UK Biobank [17] and ABCD [18] datasets were included in the analysis. All ENIGMA cohorts and UK Biobank mainly consisted of adult participants, and ABCD was a youth cohort. Demographic information for each individual cohort can be found in Supplementary Table 1. All cohorts obtained approval from their local institutional review boards and ethics committees. Written consent was obtained from all participants and their caregivers if the participants were minors. The study was approved by the NHS Tayside Research Ethics committee (05/s1401/89). Ethics approval for each individual cohort can be found in their protocol papers and in Supplementary Materials (Supplementary Methods section).

### Summary statistics for MD GWAS

This study used Howard et al. (2019) MD GWAS summary statistics [3], excluding 23andMe and all overlapping individuals with the ENIGMA/UK Biobank imaging/ABCD datasets. Although MD is a broad trait that include Major Depressive Disorder, it showed high genetic correlation with clinically ascertained Major Depressive Disorder (rG = 0.86) [3]. Specifically, potentially overlapping individuals between the GWAS and testing samples were identified and then removed using the Psychiatric Genetic Consortium (PGC) CheckSums algorithm [3]. The CheckSums algorithm is an established algorithm that can help identify overlapping samples anonymously. An analysis proposal regarding this study was approved by PGC to allow removing potential overlapping samples. The CheckSums scripts were sent to all the ENIGMA cohorts and the outputs were then shared with the PGC GWAS analyst. Therefore, no ENIGMA study had access to the CheckSums data of PGC or of any other ENIGMA study. Participants that were both part of the GWAS and the ENIGMA Major Depressive Disorder consortium were removed from the discovery GWAS, and an updated analysis of the summary statistics was conducted by a PGC analyst. ABCD study was not included in the MD GWAS as the age group was significantly younger than all participants in the GWAS and therefore no CheckSums analysis was needed. The final MD GWAS summary statistics covered 727,742 individuals. These MD GWAS summary statistics were subsequently used to calculate MD PRS in independent participants.

### Genetic data processing

**Fig. 1:**
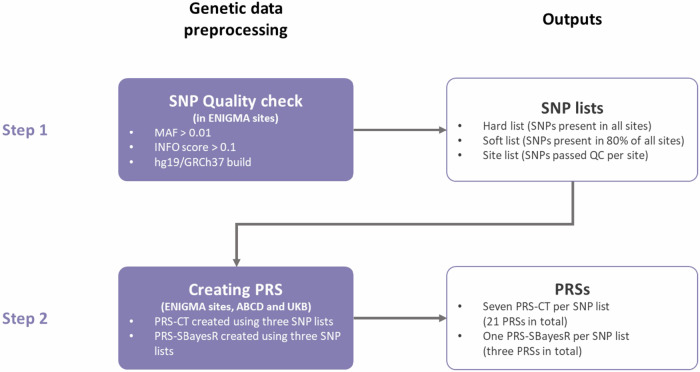
Flowchart of genetic data processing. PRS processing was performed in two steps. The first step contained standardised quality check for genetic data and SNP selection. Specific criteria are summarised in the figure and detailed in the Methods. The outputs from the first step were then used for the second step of generating PRS for each individual cohort. All PRS created are summarised in the figure and detailed in the Methods. QC quality check, MAF minor allele frequency, PRS polygenic risk score, PRS-CT PRS created using clumping and thresholding method, PRS-SBayesR PRS created using SBayesR.

Quality check (QC) and pre-processing were conducted locally by each individual cohort providing access to linked genetic and imaging data, with only anonymised individual-level PRS data being shared for analyses (Fig. 1). Hard-call, imputed genetic data were used for generating PRS. Selection of SNPs included in creating PRS was performed in a two-stage manner. First, all ENIGMA cohorts shared a list of SNPs in the hg19/GRCh37 build that passed the QC criteria of minor allele frequency (MAF) > 0.01 and INFO score > 0.1. Based on these SNP lists, we used three lists of SNPs for each individual cohort to create PRS: (1) a hard list: SNPs present in all ENIGMA cohorts (N_SNP_ = 3,176,977), (2) a soft list: SNPs present in more than 80% of the cohorts in ENIGMA (N_SNP_ = 6,306,997) and (3) a cohort list: all SNPs that passed QC for the specific individual cohort (N_SNP_ varies per cohort). These three lists were also applied to select SNPs in the UK Biobank and ABCD. For cohort lists in the UK Biobank and ABCD, a lower threshold of minor allele frequency (MAF > 0.001) was used due to the large sample sizes of these cohorts.

### Calculation of PRS for MD

We created PRS for MD using two methods. The first method we used was the clumping and thresholding method (PRS-CT) [19]. We created seven PRS-CT using PRSice 2.0 [19] based on seven p-value thresholds (pT = 5 × 10^−8^, 1 × 10^−4^, 1 × 10^−3^, 0.01, 0.1, 0.5 and 1). Clumping was conducted using a window of 500 kb with a r^2^ < 0.1. For smaller studies with *N* < 1000, genotype data of central European samples (CEU) from the 1000 Genomes Project was used as reference panel for clumping [20]. For larger studies of *N* > 1000, their own imputed genetic data was used as reference data for clumping.

We also created a second set of PRS using a Bayesian method (PRS-SBayesR) [21]. The Bayesian method of creating PRS has become increasingly used due to its better predictive power. MD summary statistics were processed using ‘SBayesR’ [21], and the shrunk sparse LD matrix that covers 2.8 million common SNPs was used as the LD reference panel [21]. Markov chain Monte Carlo (MCMC) chain length was set as 21,000. The output summary statistics of SBayesR were then used for creating PRS. A total of 86,787 SNPs with non-zero effect sizes were included in the SBayesR summary statistics. The summary statistics were then used in PRSice 2.0 to create the PRS, and no clumping was applied. As a result, six sets of PRS were generated per cohort (three SNP lists × two PRS methods). Scripts are available at the URL: https://github.com/xshen796/ENIGMA_mdd_prs/blob/main/script/PREP_PRS/Calculate_PRS.md.

### Neuroimaging measures

T1-weighted images were obtained and pre-processed locally for each individual cohort [5, 7]. Anonymised individual-level FreeSurfer outputs were shared for analysis. The T1-weighted images were preprocessed, quality-checked and parcellated with FreeSurfer version 5.0 or 5.3 using the ENIGMA3 – GWAS Meta Analysis of Cortical Thickness and Surface Area protocol. Detailed description can be found in the protocol paper [22] and in the URL: https://enigma.ini.usc.edu/protocols/imaging-protocols/.

**Fig. 2:**
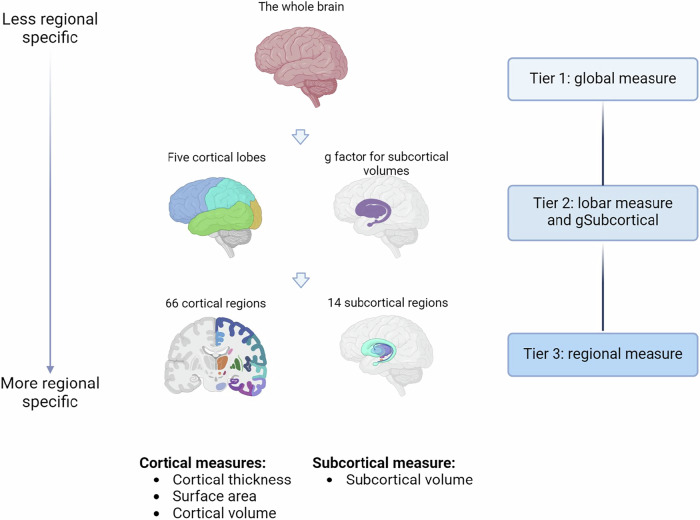
Neuroimaging measures tested. Associations between MD PRS and brain structural measures were tested hierarchically in three tiers. Tier 1 includes global measures, tier 2 consists of lobar structural measures (cortical thickness and surface area) or the entire subcortical structure, and finally, tier 3 consists of regional measures (cortical thickness, surface area and subcortical volume) of each individual region.

The neuroimaging measures were generated in a three-tier hierarchical order [23] (see Fig. 2): (1) global measures, (2) lobar measures and g factor of subcortical volumes (gSubcor) and (3) regional measures. The global measures include intracranial volume (ICV), global average cortical thickness and total brain surface area. The lobar measures included cortical thickness and surface area estimates of five lobes: temporal, parietal, occipital, frontal and cingulate lobes. Cortical thickness and surface area for each lobe was calculated by extracting the mean of thickness and the sum of surface area for all regions that lie within the lobe. For each participant, lobar measures which had more than two regions with missing values were set as ‘NA’ and therefore removed from further analysis. Definitions of lobar regions can be found elsewhere [23] and in the Supplementary Materials. The gSubcor [6] measure represents the score on the first unrotated principal component (PC), derived with PCA conducted on all subcortical volumes. The first unrotated PC explains 64.9% of the total variance of all 14 subcortical volumes (Supplementary Fig. 1). Finally, regional measures included cortical thickness and surface area estimates of 33 bilateral cortical regions defined by the ‘Desikan-Killiany’ cortical atlas [5, 24] (66 measures per participant) as well as subcortical volumes for 7 regions defined by the ASEG subcortical atlas (14 measures per participant) [7, 25]. Lateral ventricles and the temporal pole were not included in the analyses, as these measures were not available in large cohorts (e.g. UKB, see Supplementary Materials).

### MD PRS association analysis

#### Validation of MD PRS prediction

To validate the MD PRS, out-of-sample prediction of all the MD PRS (eight PRS in total, including seven PRS-CT and one PRS-SBayesR) was performed using a mixed-effect linear regression model [26]. Before entering the linear regression model, each MD PRS was residualised against covariates to account for genetic relatedness and population stratification. Different strategies were applied separately for ENIGMA cohorts, the ABCD study and UK Biobank. For ENIGMA cohorts, MD PRS were regressed against genomic relationship matrices. Genomic relationship matrices were derived using the imputed genetic data that passed quality check using GCTA [27], and they were fit as the random factor. The residualised scores were generated per cohort using the ‘lmekin’ function from the ‘coxme’ R package [28]. For the ABCD study, MD PRS were regressed against genetic principal components, batch, family ID, assessment site and an additional term of ‘family ID|assessment site’ to reflect the nested structure, as per recommendation by the ABCD team [29]. A mixed-effect, linear model was used for the ABCD study, using the ‘lmer’ function from the ‘glmer’ R package. Family ID, assessment site and the nested term were included as random factors, and genetic principal components were set as fixed factors. For UK Biobank, because its genomic relationship matrix was particularly computationally demanding to handle, we regressed the MD PRS against a kinship relationship matrix using a similar approach that was implemented in our previous work [30]. Kinship relationships up to second-degree relatives were derived using the ‘King’ software [31]. The first ten 10 genetic principal components, genotyping array, scanner site and head positions in the scanner on the x-, y-, z- and table axes [32] were included as additional covariates along with the kinship matrix. Residualised scores for UK Biobank were obtained using the restricted maximum likelihood approach in GCTA [27]. The scores were set to match the standard deviation of the original uncorrected scores so as to keep the scores within the consistent scale with other studies. The above correction of technical confounders and relatedness were performed on each individual site. The corrected data was then merged as one single dataset for further analyses. Correlations between PRS and PRS residualised against covariates for each individual study were reported in Supplementary Table 2 and Supplementary Fig. 2.

Mixed-effect logistic regression was used to test the association between the residualised PRS and MD using the ‘glmer’ function in the ‘lme4’ R package [33], adding age and sex as fixed-effect covariates and cohort as a random factor. MD case-control diagnosis was set as the dependent variable (y) and MD PRS were set as the independent variable (x). Standardised log-transformed odds ratios (OR) were reported as effect sizes.

#### Association between MD PRS and neuroimaging measures

All analyses on associations between MD PRS and brain structural measures were conducted in the full sample first, followed by additional analyses conducted in youth samples (individuals ≤25 years old across all cohorts).

Associations between MD PRS and neuroimaging measures were tested using mixed-effect linear models similar to the model used for MD PRS prediction (‘lmer’ function in the ‘lme4’ R package was used). All the covariates and genomic/kinship relationship matrix and technical covariate correction were kept consistent with the MD PRS prediction analyses. Neuroimaging measures were set as the dependent variables and MD PRS as the independent variables. Age and sex were added as fixed-effect covariates and site was added as a random-effect covariate in the linear model.

Associations with MD PRS were tested separately for global, lobar and regional measures (see Fig. 2). PRS which were most strongly associated with global measures amongst all PRS-CT and PRS-SBayesR were further analysed for associations with lobar and regional measures. When regional measures were analysed, an additional model that included ICV as a covariate was tested as secondary analysis to see if the regional effect was over and above global volume. An additional sensitivity analysis was performed to investigate the interaction of MD PRS and MD case-control status in the full sample.

Standardised regression coefficients (β) were reported as effect sizes. P-values were FDR-corrected for lobar and regional measures per type of structural measure (e.g. p-values for cortical thickness were corrected across all 66 regions, and p-values for subcortical volumes were corrected across all 14 regions). We thus refer to this correction as whole-brain FDR correction. We further applied two additional methods of multiple testing correction, which were reported in the Supplementary Materials. The first multiple testing correction method was Bonferroni correction per type of structural measure (i.e. whole-brain Bonferroni correction), and the second method was FDR correction across all measures tested (146 regions in total, i.e. all-measure FDR correction). For global measures, FDR-correction was applied across all PRS and global measures (eight PRS × three global measures).

To investigate specific associations in younger people, we conducted a separate set of association analyses in young individuals (<25 years old). A total of 5570 young subjects from seven cohorts were included in the analyses (see demographic information in Supplementary Table 1). To formally test the concordance of effect sizes across regions between the associations found in youth and in the full sample, we performed (1) Pearson’s correlation analysis on the effect sizes of regional structural measures, and (2) the exact binomial test of directions of effects for regional measures using the ‘binom.test’ function from the ‘stats’ R package (‘−1’ represents a negative effect size and ‘+1’ represents a positive effect size, hypothesised random probability of success set as 0.5).

### Mendelian randomisation (MR)

To investigate the potentially causal relationship between brain structure and MD, we conducted bi-directional MR for the brain structural measures that showed significant association with MD PRS. MR utilises genetic variants as instruments for testing causality, and has been particularly useful for identifying causal biomarkers for diseases [34].

#### MD GWAS

We used the summary statistics from the meta-analysis of the MD GWAS for creating PRS (non-imaging sample of UKB, PGC cohorts) and summary statistics of European subjects from 23andMe, Inc [3]. For the additional summary statistics from 23andMe, participants provided informed consent and volunteered to participate in the research online, under a protocol approved by the external AAHRPP-accredited IRB, Ethical & Independent (E&I) Review Services. As of 2022, E&I Review Services is part of Salus IRB (https://www.versiticlinicaltrials.org/salusirb). The full GWAS summary statistics for the 23andMe discovery data set will be made available through 23andMe to qualified researchers under an agreement with 23andMe that protects the privacy of the 23andMe participants. Datasets will be made available at no cost for academic use. Please visit https://research.23andme.com/collaborate/#dataset-access/ for more information and to apply to access the data.

#### GWAS for neuroimaging traits

GWAS summary statistics of neuroimaging traits were extracted from the Smith et al. study of ~33,000 participants with European ancestry. Details of the study can be found elsewhere [3, 4]. The discovery GWAS sample for neuroimaging traits had no overlap with the MD GWAS, which was ensured by using the same protocol for the PRS association analysis. Neuroimaging traits which demonstrated an association with MD PRS and had >10 instrumental genetic variants (IVs) after clumping were included in the MR analysis.

#### MR data preparation and analysis

We used the ‘TwoSampleMR’ R package (version 0.5.6) for all MR analyses. Data preparation included standard procedure described elsewhere [4]. In brief, GWAS summary statistics of exposure variables went through QC, and variants were retained if MAF > 0.01, INFO score>0.1, and Hardy-Weinberg equilibrium p-value < 1 × 10^−5^. Exposure summary statistics were then clumped using a r^2^ < 1 × 10^−3^ with a 1 MB window using the default European LD reference panel. Clumping was performed using the ‘clump_data’ function from the ‘TwoSampleMR’ package. After quality check and clumping, MD had 122 IVs retained as exposure data, and the number of IVs for neuroimaging traits ranged from 10–24 (median number of IVs = 15).

Three methods were used for bidirectional MR: (1) inverse variance weighted (IVW, the main method), (2) weighted median, and (3) MR Egger (with bootstrapping). For the MR analysis of causal effect of MD, due to the large number of IVs of MD, we used the ‘contamination mixture’ method as a secondary analysis in addition to IVW. The contamination mixture method has a particular advantage when handling a large number of IVs, and typically results in low Type I error rates when the numbers of invalid IVs are small. Horizontal pleiotropy and heterogeneity of IVs were estimated using Egger intercept and Q statistics, respectively. Those associations were identified as potentially causal association if: (1) IVW/contamination mixture test was FDR-significant, (2) confirmed by nominally significant weighted median results in the same direction of IVW and (3) nominally significant MR Egger analysis if there was any horizontal pleiotropy indicated by an Egger intercept significantly deviating from zero.

## Results

### Validation of MD PRS

PRS created using the three sets of SNPs were highly correlated (all r > 0.99). Therefore, all the following analyses were conducted using PRS created using only the ‘cohort list’ variant that was based on SNPs that passed QC criteria for each individual cohort.

For the PRS-CT, those PRS which were created using p-value thresholds higher than 0.001 were associated with MD diagnosis (log-transformed odds ratio ranged from 0.041–0.162, p ranged from 0.002 to 1.5 × 10^−34^, Supplementary Table 3). The PRS-SbayesR had stronger association than all the PRS-CT (β = 0.181, *p* = 2.83 × 10^−42^).

MD prediction for each individual study cohort can be found in Supplementary Fig. 3.

### Association between MD PRS and global neuroimaging measures

**Fig. 3:**
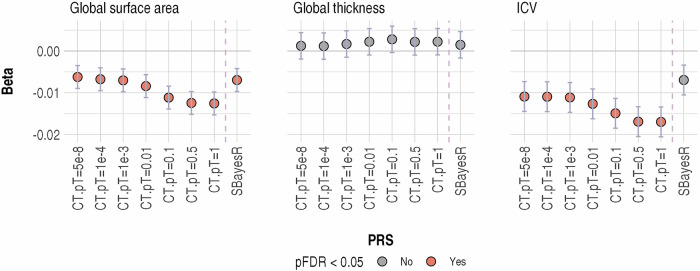
Association between all MD PRS and global neuroimaging measures. X-axis represents individual PRS. CT clumping and thresholding method, pT p-value threshold used for creating PRS using clumping and threshold methods, and SBayesR PRS created using the SBayesR-processed summary statistics. Y-axis represents β, i.e., standardised regression coefficient.

Higher MD PRS were associated with lower ICV for all the PRS-CT (β ranged from −0.011 to −0.017, p ranged from 0.002–1.97 × 10^−6^, pFDR < 0.005, see Fig. 3, Supplementary Fig. 4 and Supplementary Table 4). The effect size for association between ICV and PRS-SBayesR was in the same direction but did not reach FDR-corrected significance (β = −0.007, *p* = 0.052, pFDR = 0.078). All PRS-CT and PRS-SbayesR were also associated with lower total cortical surface area (β ranged from −0.006 to −0.013, p ranged from 0.023–4.5 × 10^−6^, pFDR<0.036). No association was found between any MD PRS and global cortical thickness (β ranged from 0.001–0.003, *p* > 0.377).

Since MD PRS-CT created at the p-value threshold of 1 (pT = 1) showed the strongest association with both ICV (β = −0.017, *p* = 1.97 × 10^−6^) and global cortical surface area (β = −0.013, *p* = 4.5 × 10^−6^), analyses for this MD PRS was carried on for all regional measures.

### Association between MD PRS-CT (pT=1), lobar measures and gSubcortical

Lower surface areas were associated with the MD PRS-CT at pT = 1 in all five lobes (β ranged from −0.006 to −0.011, p ranged from 0.033–2.85 × 10^−6^, pFDR <  0.033, see Supplementary Table 5). The strongest association was found with surface area in the frontal lobe (β = −0.011, *p* = 2.85 × 10^−6^, pFDR = 1.42 × 10^−5^). Lower gSubcortical was also found associated with the MD PRS (β = −0.011, *p* = 4.41 × 10^−4^).

No lobar measure of cortical thickness was associated with MD PRS-CT at p-T = 1 (absolute β ranged from 0.002–0.005, *p* > 0.149).

### Association between MD PRS-CT (pT=1) and regional cortical thickness and surface area

**Fig. 4:**
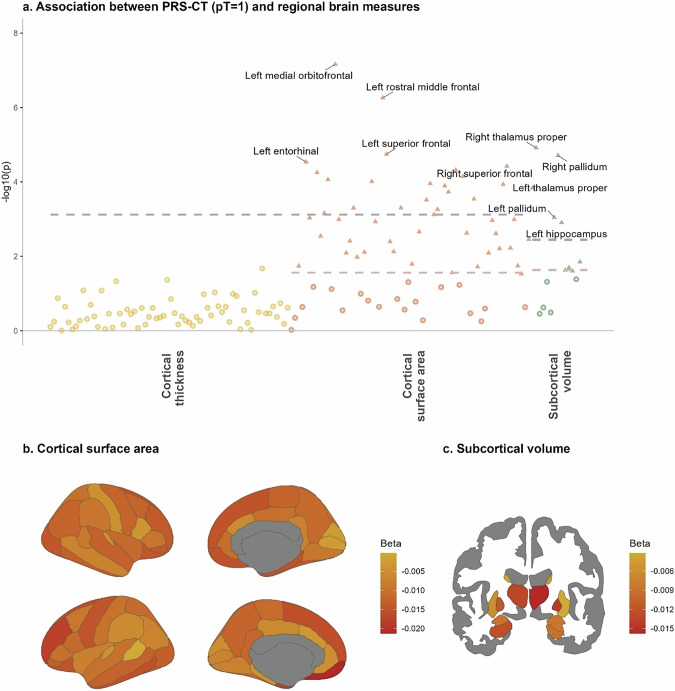
Association between PRS-CT at pT=1 and brain regional measures. **a** P-value plot for associations between MD PRS and all regional measures. X-axis represents the three categories of brain regional measures. Y-axis represents -log10-transformed p-values. Each dot represents the result for one brain regional measure. Red and grey dashed lines are the FDR- and Bonferroni-significance threshold, respectively. Associations that are significant after FDR-correction are highlighted as solid dots. Top five FDR-significant associations per category are annotated with labels of regions in the figure. Multiple-comparison correction was applied within each individual category. No associations were found in cortical thickness and therefore only the Bonferroni-significance threshold is shown in the figure. **b**. Regional results for cortical surface areas. Darker colours represent lower beta-values (standardised regression coefficients). **c**. Regional results for subcortical volumes in coronal view.

Without correction for ICV, cortical surface areas in 46 regions (out of 66 regions tested) were associated with MD PRS-CT at pT = 1 and reached FDR-significance (β ranged from −0.009 to −0.021, p ranged from 0.031–9.48 × 10^−8^, pFDR <  0.044). Using a more stringent FDR correction across all measures tested, 39 regions remained significant after multiple testing correction (β ranged from −0.01 to −0.021, p ranged from 0.01–9.48 × 10^−8^, pFDR across all measures < 0.035). Within the 46 FDR-significant regions, 34 were bilateral regions that had associations in both hemispheres (17 regions of both hemispheres), and 12 regions had associations in one hemisphere only. Strongest associations were found in the medial orbito-frontal gyrus in both hemispheres (left hemisphere: β = −0.021, *p* = 9.48 × 10^−8^, pFDR = 6.26 × 10^−6^; right hemisphere: β = −0.015, *p* = 5.19 × 10^−5^, pFDR = 5.83 × 10^−4^) and in bilateral superior frontal gyrus (left hemisphere: β = −0.016, *p* = 2.4 × 10^−5^, pFDR = 5.29 × 10^−4^; right hemisphere: β = −0.015, *p* = 5.3 × 10^−5^, pFDR = 5.83 × 10^−4^). See Fig. 4, Supplementary Fig. 5 and Supplementary Tables 6, 7 for regional statistics.

A secondary analysis was carried out on surface areas measures after controlling for ICV (Supplementary Fig. 6). Focusing on the measures that reached FDR-significance in their associations with MD PRS-CT, left medial orbito-frontal gyrus remained significant after controlling for ICV (β = −0.011, *p* = 0.001, pFDR = 0.048). No other regions that were previously associated with MD PRS-CT reached FDR-corrected significance after controlling for ICV (*p* > 0.003, pFDR > 0.077).

No association was found between regional cortical thickness and MD PRS-CT (pT = 1) (absolute β ranged from 1.96 × 10^−4^ to 0.008, *p* > 0.023, pFDR > 0.7).

### Association between MD PRS (pT=1) and subcortical volumes

Without correction for ICV, there were five subcortical regions that showed FDR-significant associations with MD PRS-CT (pT = 1), out of the 14 regions tested (β ranged from −0.011 to −0.015, p ranged from 0.002–1.73 × 10^−5^, pFDR <  0.006, see Supplementary Table 8). All five subcortical regions remained significant using a more stringent FDR-correction across all regional measures (pFDR across all measures <  0.01). Out of all the FDR-significant regions, lower volumes of the thalamus and pallidum were associated with higher MD PRS-CT bilaterally (β ranged from −0.011 to −0.015, p ranged from 0.001–1.73 × 10^−5^, pFDR < 0.004). Lower volume of the left hippocampus was associated with higher MD PRS (β = −0.012, *p* = 0.002, pFDR = 0.006). After controlling for ICV in the association model, nominally significant association was found in the right pallidum (β = −0.006, *p* = 0.042, pFDR = 0.197), but no other associations were found (*p* > 0.079).

### Interaction between MD PRS and MD case-control status

No interaction between MD PRS and MD case-control status reached FDR-significance in the entire sample for any brain measure (*p* > 0.028, pFDR > 0.96).

### Analysis specifically in youth (≤25 years old)

In addition to the main analyses conducted on the entire sample, we conducted additional analyses on young individuals ≤ 25 years old (*N* = 5570 from seven cohorts, see Supplementary Table 1). For global neuroimaging measures, no association reached FDR-corrected significance (for all cortical measures: absolute β ranged from 2.75 × 10^−4^ to 0.022, *p* > 0.071). Similar to the adult sample, effect sizes were larger for ICV and cortical surface area than cortical thickness (see Supplementary Fig. 4 and Supplementary Table 4). No association was found in lobar/gSubcortical measures (absolute β ranged from 3.55 × 10^−4^ to 0.013, *p* > 0.266, Supplementary Table 5). However, direction of associations for ICV and global surface area were consistent with associations found in the entire sample (β ranged from −0.007 to −0.022 for ICV, and β ranged from −2.75 × 10^−4^ to −0.011 for global surface area).

For regional neuroimaging measures, we did not find any associations that reached FDR-corrected significance (absolute β ranged from 5.18 × 10^−5^ to 0.037, *p* > 0.004, pFDR > 0.498, see Supplementary Tables 6–8). The strongest association was found between thickness in the right posterior cingulate gyrus and PRS-CT at pT = 1 (β = 0.037, *p* = 0.004, pFDR = 0.271). Although no association reached FDR-significance, effect sizes for regional cortical surface areas were positively correlated between the youth and the full sample (r = 0.43, *p* = 3.21 × 10^−4^), and the directions of effects were significantly consistent (71.2% associations in the same direction, exact binomial test p-value = 7.56 × 10^−4^). This pattern was seen to a lesser degree in cortical thickness, for which effect sizes in youth and the full sample showed a significant but lower correlation (r = 0.34, *p* = 0.006), and the exact binomial test suggested that concordance of direction of association was not significantly higher than chance (56.1% associations in the same direction, exact binomial test p-value = 0.389). Similarly, a low concordance rate of direction of association was found for subcortical volumes between the youth and full sample (correlation of effect sizes: r = 0.752, *p* = 0.002; 42.9% associations in the same direction, exact binomial test p-value = 0.791).

### MR

**Fig. 5:**
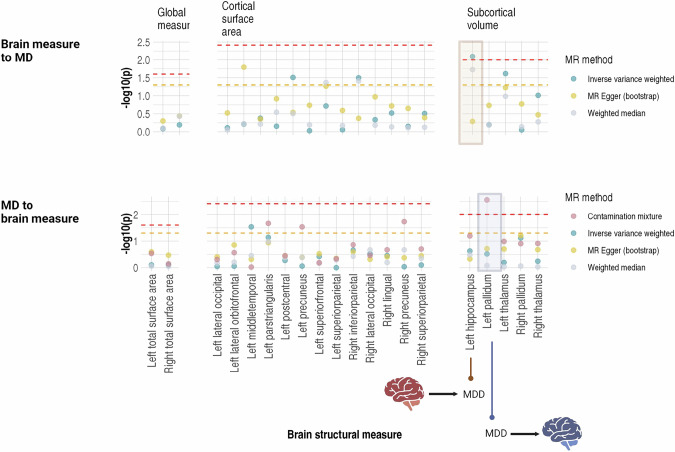
Results of the bi-directional Mendelian randomisation analysis for the causal relationships between MD and neuroimaging traits. The top panel shows the results of causal effects of brain structural measures to MD, and the bottom panel shows the causal effects in the reverse direction. X-axes represent individual brain structural measures, and the measures were separated by their category (global measure/cortical surface area/subcortical volume). Y-axes represent -log10-transformed p-values. Neuroimaging traits were highlighted in the plot if there was any potentially causal effect that reached FDR-significance indicated by the inverse variance weighted method.

We identified a potentially causal effect of smaller left hippocampal volume on higher liability for MD (see Fig. 5, Supplementary Fig. 7, Supplementary Tables 9, 10). Left hippocampal volume showed a significant effect on MD in IVW analysis (β = −0.064, *p* = 8.04 × 10^−3^, pFDR = 0.04), supported by a nominally significant weighted median result (β = −0.049, *p* = 0.019). MR Egger analysis was not significant (β = −6.33 × 10^−4^, *p* = 0.514), however, no horizontal pleiotropy was observed (p-value for Egger intercept = 0.346). On the contrary, there was no causal effect identified from MD to left hippocampal volume (*p* > 0.064 for all MR methods, Supplementary Data 1).

No causal relationship in the reverse direction (from MD to neuroimaging traits) was FDR-significant, as assessed with IVW (*p* > 0.073, pFDR > 0.379) or weighted median method (*p* > 0.104, pFDR > 0.684). The only causal effect found from MD to brain structural trait was in the left pallium using the contamination mixture method (β = −0.179, *p* = 0.003, pFDR = 0.014, No. of valid IVs = 89).

## Discussion

We used a large-scale mega-analysis on individual-level data and found that higher PRS for MD were associated with smaller global cortical surface area, lower intracranial volume and lower subcortical volumes, but not cortical thickness. Further analysis of lobar measures indicated that the largest difference associated with MD PRS was in the surface area of the frontal lobe. More specifically, smaller cortical surface areas in regions including the medial orbito-frontal gyrus, superior frontal gyrus and temporal lobe, were associated with higher MD PRS. In subcortical regions, lower volumes of the thalamus, pallidum and hippocampus were found associated with higher PRS for MD. In young individuals below 25 years old, no associations survived correction for multiple comparison in the relatively small size of the subsample. However, directions of the effects remained similar to those found in the analyses of the entire study sample.

Our study identified brain structural measures that were previously found to have case-control differences in MD, for example, ICV and hippocampal volume [7]. However, the majority of brain structural associations identified in our analysis had not been found consistently in case-control studies. A major difference is that our findings mainly fell within the category of cortical surface areas, whereas cortical thickness differences are more commonly reported in adult MD case-control studies [5]. Our findings of differences in cortical surface areas are, however, supported by findings from studies of genetic correlation [22, 35]. For example, genetic correlations with MD were observed with cortical surface areas, and the associations were stronger than with cortical thickness [22]. Such pattern of stronger association between MD and surface areas than cortical thickness was also observed in youths [36]. The fact that differences were found in surface areas rather than cortical thickness may imply that genetic predisposition to MD may manifest particularly prominently early in life, while abnormality in either cortical thickness or surface area may manifest in different age groups across the life course [37, 38]. Various reasons could contribute to differences in associations of PRS and or diagnosis of MD with the brain, including varying environmental influence across the life course at critical timepoints [35]. Future studies are needed to further explore the gene-by-environment interaction that may contribute to these differences.

One of the strongest regional associations was found in the surface area of the medial orbitofrontal cortex (OFC), and this association remained significant after controlling for ICV. The differences in both structure and functional activity of the OFC in MD has been consistently documented [5, 37]. Structural differences in OFC were previously found to be associated with depressive symptoms in treatment-naïve patients [38] and in pre-school youths [39], suggesting that impairment in OFC may be particularly important in early development of depressive symptoms. Participants with brain lesions in OFC showed lower level of depressive symptoms compared to those with lesions in other brain areas [40]. Moreover, brain stimulation of the OFC area improved mood state in MD patients [41]. These studies suggest a potential causal role of changes in the OFC in MD. Brain activity in OFC is associated with known protective factors for MD, such as reward processing [42, 43] and regulation of stress caused by negative social interactions (e.g. social exclusion) [44, 45]. Anatomically, the lower medial wall of the PFC (medial OFC according to the Desikan–Killiany atlas in FreeSurfer) contains the subgenual anterior cingulate cortex and subcallosal gyrus that serve as homeostasis sensors and control the HPA axis responses through connections to the hypothalamus [46]. Taken together, the functional and structural findings indicate that delayed or abnormal development of OFC may manifest in mood regulation (e.g., reward processing) impairments that are prevalent in MD patients.

In subcortical regions, lower hippocampal volume was previously found associated with lifetime and current MD, and the association was particularly strong in patients with recurrent episodes and in patients with early age of onset < 21 years old [7]. Our findings confirm the crucial role of the hippocampus in MD, as well as its potentially causal relationship with MD [47–49]. Findings from early life adversity and its influence on hippocampal structural integrity also speak to a potentially causal pathophysiological role of hippocampal deficits for the liability to develop an affective disorder [50]. As such, both genetics and imaging studies provided a potentially causal relationship from hippocampal volume to MD. Further, we report a novel association between PRS for MD and thalamic volume. A previous study looking at genetic correlations found that thalamic volume had overlapping genetic architecture with MD and the genetic correlation was stronger for MD compared with other major psychiatric disorders (e.g. schizophrenia) [35]. Previous large-scale studies found that MD and higher PRS of MD were associated with lower integrity in white matter microstructure of thalamic radiations [4]. In addition to findings related to structural variations, three independent functional MRI studies showed that thalamic activity may be an indicator for treatment outcomes [51–53]. The thalamus is an important hub that connects the fronto-parietal network with the limbic system [54]. Considering its specific anatomical importance in the structural connectome, associations with MD indicate that there is an overall turbulence of thalamic structural/functional network in the disorder [54]. Further studies are needed to directly examine the role of the thalamus and its disruption in the greater neural network.

We are the first to conduct a mega-analysis to investigate associations between genetic risk of MD and brain structure. We took advantage of a large multi-site sample and adopted a hypothesis-free, data-driven approach. This strategy enabled us to reveal novel associations that were not detected in smaller studies, which typically used a region-of-interest approach. A few important sensitivity analyses were conducted to estimate the extent to which heterogeneity of data pre-processing might contribute to between-site differences. In particular, we found that there was minimal difference between the PRS generated using locally quality-checked, site-specific SNP lists and the PRS generated using a single consistent list across sites. Our study is also one of the first to evaluate the influence of different lists of SNPs between cohorts and to implement rigorous examination of sample overlap between training and testing samples in genetic imaging studies. Our findings and the applied methods provide important technical guidance for future neuroimaging-genetic studies and may be used for studying other brain phenotypes, such as those based on functional imaging and diffusion tensor imaging. However, it is important to note that MD is well-known as a highly polygenic trait, and datasets included in the present study were relatively large (all *N* > 200). It is, therefore, unlikely that heterogeneity of SNPs between studies may have had major impact on the results. Caution should be taken especially when considering traits with more sparse genetic architecture influenced by certain genetic variants with relatively stronger effect sizes. And thus inconsistent availability in these genetic variants may pose stronger influence on the heterogeneity in PRS.

The current study has a few limitations. First, we looked only at European samples due to the population constrained GWAS used for creating PRS. It has been found that trans-ancestry PRS tend to have compromised statistical power due to the confounding effect from heterogeneous genetic architectures between ancestry groups [55]. It is imperative that further studies aim to extend the analysis to other ancestry groups when well-powered, non-European GWAS for MD becomes available. More accurate imputation techniques may help maximise the use from samples that have mixed ethnic backgrounds [56]. Second, we utilise MD PRS as key measure since it includes both self-reported and clinically ascertained Major Depressive Disorder. MD is a broader definition of depression, capturing the self-reported core symptoms of MDD, including persistent and severe low mood or despair. MD PRS has demonstrated statistical power to differentiate both self-reported and clinically ascertained cases from controls [3]. Further, multiple versions of FreeSurfer may introduce biases to the results. However, this issue is unlikely to affect larger studies included in our analysis (e.g. UKBB and ABCD). Finally, the young samples in the present study are relatively small which limits the statistical power to find reliable associations in this age group. Although we included participants from a wide age range, participants in early adulthood were underrepresented [16]. Future studies may benefit from focusing on younger participants and studying disease progression into later life [35].

In conclusion, our analysis identified novel associations between PRS for MD, global cortical surface area and intracranial volume. Genetic risk of MD was, in particular, associated with lower surface area in OFC, and lower hippocampal and thalamic volumes. It was not, however, associated with differences in cortical thickness. For the hippocampus, MR analysis demonstrated a causal effect from hippocampal volume to MD. Previous meta-analysis did not report MD case-control differences in surface areas [5], but our findings are convergent with those of a previous study of genetic architecture of longitudinal brain structural changes [35]. Future studies are necessary to investigate longitudinal changes in the cortical regions identified here, and to define the effects of gene-by-environment interactions on these regions.

## Supplementary information

Supplementary Materials

Supplementary Data 1

## Data Availability

GWAS summary statistics for Major Depression are publicly available at the URL: https://datashare.ed.ac.uk/handle/10283/3203. The full GWAS summary statistics for the 23andMe discovery dataset will be made available through 23andMe to qualified researchers under an agreement with 23andMe that protects the privacy of the 23andMe participants. Datasets will be made available at no cost for academic use. Please visit https://research.23andme.com/collaborate/#dataset-access/ for more information and to apply to access the data. GWAS summary statistics for brain structural measures are available at the Oxford Brain Imaging Genetics Server (https://open.win.ox.ac.uk/ukbiobank/big40/). According to the terms of consent, access to any form of individual-level data requires to approval and consent from each individual cohort.
