# Supplementary material for: Association between polygenic risk for Major Depression and brain structure in a mega-analysis of 50,975 participants across 11 studies
Source: Mol Psychiatry. 2025 Aug 19;31(2):611–21. doi: 10.1038/s41380-025-03136-4 (PMC12815664; doi:10.1038/s41380-025-03136-4)
Supplement: Supplementary file 1 — Supplementary Materials [file 41380_2025_3136_MOESM1_ESM.docx]

Supplementary Materials

Association between polygenic risk of Major Depression and brain structure

Shen X. et al.

#####

## Supplementary Methods and Results

### Genome-wide association study for Major Depression

In the GWAS for Major Depression (MD) by Howard et al.^1^, summary statistics from UK Biobank, 23andMe and Psychiatric Genomics Consortium (PGC) were meta-analysed. MD was defined based on self-declared medical visits or depressive status in UK Biobank and 23andMe. GWAS from PGC was primarily based on clinical structured interview or questionnaires.

UK Biobank: Used a broad depression definition based on self-reported visits to healthcare professionals. Cases were identified through two questions: whether participants had ever seen a GP or psychiatrist for "nerves, anxiety, tension or depression." Individuals with bipolar disorder, schizophrenia, personality disorder, or those on antipsychotic medications were excluded.

23andMe: Depression status was determined through web-based surveys where individuals self-reported receiving a clinical diagnosis or treatment for depression.

PGC: The study focused on clinically-derived phenotypes for MDD from 29 cohorts, based on established international diagnostic criteria (DSM-IV, ICD-9, or ICD-10), confirmed through structured clinical interviews, professional assessments, or medical records^2^.

### Controlling for relatedness

The approach of regression against genomic relationship matrices (GRM) or kinship matrices has been previously applied in PRS studies, especially when sample sizes are large^3-6^. By regressing out the GRM/kinship matrices, we were able to control relatedness per cohort, while at the same time reduce the computational burden to a manageable scale. The ENIGMA studies were much smaller (N < 3000), and thus it was possible to control for GRM. However, ABCD and UKB have considerably larger samples (N > 8K) and pose great challenge to regressing against GRM (> 1T ram may be required per model). However, regressing against kinship matrix was significantly more computationally manageable (~ 256G ram required). Although this approach was less ideal, regressing out kinship matrix has been commonly demonstrated as being able to sufficiently account for relatedness^7^. Thus, we applied this approach of regressing against GRM for smaller cohorts (N < 3000), and for the bigger cohorts, we regressed PRSs against kinship matrices or equivalent (e.g. controlling for family structure).

**Selection of PRS for association analyses with brain structural measures**

We chose the PRS with strongest association with global brain measures due to known discrepancies between PRSs that best predict depression versus brain anatomy^8, 9^. While PRS at p=1 and SBayesR-derived PRS showed similar effect sizes for MDD prediction, the SBayesR approach uses fewer SNPs and may be more susceptible to between-cohort heterogeneity in genotyping and quality control. Therefore, we selected PRS at pT=1 for our main analyses (Supplementary Figure 10).

Distributions of PRS can be found in Supplementary Figures 11.

### Individual regions and generation of lobar measures

Cortical grey matter was divided into 66 bilateral regions (one region per hemisphere) defined by the ‘Desikan-Killiany’ atlas. The region, ‘temporal pole’, was not included in the analysis, as this region was not included in the UK Biobank imaging data, and therefore this single region has a significant smaller sample size than other regions. There were 14 subcortical regions defined by the ‘ASEG’ subcortical atlas included our analyses. Similar to the exclusion of temporal pole, lateral ventricles were not included in the analyses, as this region was not covered in the UK Biobank imaging data. Distributions of ICV and total cortical surface area can be found in Supplementary Figures 12-13.

Generation of lobar measures has been detailed in previous publication by Harris et al. (2022) in the ‘Brain imaging data’ section, Supplementary materials (URL: <https://static-content.springer.com/esm/art%3A10.1038%2Fs41398-022-01926-w/MediaObjects/41398_2022_1926_MOESM1_ESM.docx>). Measures in temporal pole were not included in generating lobar measures.

Comparison of association analyses in the global measures between UKB and ENIGMA can be found in Supplementary Figure 14. Forest plot for association analysis on global measures for each individual cohort can be found in Supplementary Figure 15.

### All-measure FDR correction

We performed a separate set of FDR correction across all measures tested at the regional level, at which different strategies of p-value correction may differ the most. FDR-correction was across all cortical thickness and surface area measures (146 cortical and subcortical measures in total). We found 45 all-measure level significant associations, which is more than what we reported using the Bonferroni correction (25 significant findings) and less than whole-brain-level significant associations (51 significant findings). For subcortical volumes, all-measure FDR correction was more lenient than both whole-brain FDR and Bonferroni corrections. We are therefore convinced that FDR correction across all-measure FDR correction does not generate significant inflation compared to whole-brain FDR correction and were not as stringent as the Bonferroni-correction we have reported in the main text (see Figure below).

**Sex*PRS interaction**

Here, we report additional analyses on PRS*sex interaction. In summary, we did not observe significant PRS*sex interaction that survived FDR correction (p_uncorrected_ > 0.028). The strongest interaction was found in cortical thickness of lingual gyrus (p = 0.028). Heterogeneity of MD may contribute to the null findings and future studies on subtypes of MD may help clarity the widely observed sex differences and its relationship with MD PRS (Supplementary Table 11-12 and Supplementary Figure 16).

### Post-hoc quality check and analysis of causal effect of left hippocampal volume on liability of Major Depression (MD)

For the significant causal effect found in hippocampal volume to liability of MDD, standard plots for quality control can be found in Supplementary Figure 7. All the IVs included in the analysis and their association statistics with the exposure (left hippocampal volume) and outcome (MD) can be found in Supplementary Table 9. Although clumping was performed on GWAS summary statistics of left hippocampal volume prior the data harmonisation and MR analysis, we ran a post-hoc quality check of the LD between IVs used for the analysis (Supplementary Figure 8). LD matrix was extracted using the 1000-Genome European samples as the reference panel (‘EUR’). The ‘ld_matrix’ function in the ‘TwoSampleMR’ R package was used to extract LD matrix.

As shown in the Supplementary Figure 8, some IV pairs showed absolute r > 0.1 (e.g. r between rs12817240 and rs4947122 = 0.12). Therefore, we conducted a separate validation analysis after removing any IV pair that had a r > 0.1 or < -0.1. To achieve this, we identified the IV pairs and retained one IV within the pair that had a stronger genetic association with hippocampal volume (lower p-value in the GWAS for left hippocampal volume). This step led to three IVs removed and the remaining 12 IVs were used in the validation analysis (Supplementary Figure 9).

Validation analysis showed consistent results as the main findings (Supplementary Tables 9-10). βs were in the same direction for all MR methods (β ranged from -0.034 to -0.042), and significant causal effect was found using IVW (β = -0.042, p = 0.024) and weighted median (β = -0.041, p = 0.044) methods. MR-Egger analysis did not reach statistical significance (β = -0.034, p = 0.255). However, there was no evidence of pleiotropy (Egger intercept = 3.65e-5, p-value for Egger intercept = 0.993).

**References**

1. Howard DM, Adams MJ, Clarke TK, Hafferty JD, Gibson J, Shirali M *et al.* Genome-wide meta-analysis of depression identifies 102 independent variants and highlights the importance of the prefrontal brain regions. *Nature Neuroscience* 2019; **22**(3)**:** 343-352.

2. Wray NR, Ripke S, Mattheisen M, Trzaskowski M, Byrne EM, Abdellaoui A *et al.* Genome-wide association analyses identify 44 risk variants and refine the genetic architecture of major depression. *Nat Genet* 2018; **50**(5)**:** 668-681.

3. Shen X, Caramaschi D, Adams MJ, Walker RM, Min JL, Kwong A *et al.* DNA methylome-wide association study of genetic risk for depression implicates antigen processing and immune responses. *Genome Med* 2022; **14**(1)**:** 36.

4. Barbu MC, Amador C, Kwong ASF, Shen X, Adams MJ, Howard DM *et al.* Complex trait methylation scores in the prediction of major depressive disorder. *EBioMedicine* 2022; **79:** 104000.

5. Walker RM, Vaher K, Bermingham ML, Morris SW, Bretherick AD, Zeng Y *et al.* Identification of epigenome-wide DNA methylation differences between carriers of APOE epsilon4 and APOE epsilon2 alleles. *Genome Med* 2021; **13**(1)**:** 1.

6. Barbu MC, Huider F, Campbell A, Amador C, Adams MJ, Lynall ME *et al.* Methylome-wide association study of antidepressant use in Generation Scotland and the Netherlands Twin Register implicates the innate immune system. *Mol Psychiatry* 2022; **27**(3)**:** 1647-1657.

7. Shen X, Caramaschi D, Adams MJ, Walker RM, Min JL, Kwong A *et al.* DNA methylome-wide association study of genetic risk for depression implicates antigen processing and immune responses. *Genome Medicine* 2022; **14**(1)**:** 36.

8. Reus LM, Shen X, Gibson J, Wigmore E, Ligthart L, Adams MJ *et al.* Association of polygenic risk for major psychiatric illness with subcortical volumes and white matter integrity in UK Biobank. *Sci Rep* 2017; **7:** 42140.

9. Shen X, Howard DM, Adams MJ, Hill WD, Clarke TK, Major Depressive Disorder Working Group of the Psychiatric Genomics C *et al.* A phenome-wide association and Mendelian Randomisation study of polygenic risk for depression in UK Biobank. *Nat Commun* 2020; **11**(1)**:** 2301.

10. Lisdahl KM, Sher KJ, Conway KP, Gonzalez R, Feldstein Ewing SW, Nixon SJ *et al.* Adolescent brain cognitive development (ABCD) study: Overview of substance use assessment methods. *Dev Cogn Neurosci* 2018; **32:** 80-96.

11. Kaufman J, Birmaher B, Brent D, Rao U, Flynn C, Moreci P *et al.* Schedule for Affective Disorders and Schizophrenia for School-Age Children-Present and Lifetime Version (K-SADS-PL): initial reliability and validity data. *J Am Acad Child Adolesc Psychiatry* 1997; **36**(7)**:** 980-988.

12. Wittchen HU. Reliability and validity studies of the WHO--Composite International Diagnostic Interview (CIDI): a critical review. *J Psychiatr Res* 1994; **28**(1)**:** 57-84.

13. Spitzer RL, Williams JB, Gibbon M, First MB. The Structured Clinical Interview for DSM-III-R (SCID). I: History, rationale, and description. *Arch Gen Psychiatry* 1992; **49**(8)**:** 624-629.

##### Supplementary Table 1. Demographic information for each individual cohort. Seven sites had a subset of individuals with age < 25 years.

| **Full sample** |  |  |  |  |  |  |  |
| --- | --- | --- | --- | --- | --- | --- | --- |
| **Cohort** | **Instruments used for diagnosis of Major depression** | **N** | **Mean age (years)** | **SD of age** | **Male (%)** | **MD (%)** | **DOI** |
| The Adolescent Brain Cognitive Development study ABCD (the ABCD study, baseline) | Structured questionnaire (KSADS) | 4,826 | 9.9 | 0.6 | 52.7 | 1.8 | 10.1016/j.dcn.2018.03.001 |
| The BiDirect study | Structured questionnaire | 914 | 50.6 | 7.6 | 45.4 | 57 | 10.1186/1471-244X-14-174 |
| The FOR2107 study | Clinical interview | 1,073 | 34.6 | 13.1 | 37 | 43.6 | 10.1007/s00406-018-0943-x |
| Generation Scotland (GS) | Structured questionnaire (SCID) | 965 | 59.7 | 10.1 | 40 | 31 | 10.12688/wellcomeopenres.15538.2 |
| Max Planck Institute of Psychiatry (MPIP) | Clinical interview (M-CIDI/SCAN) | 567 | 48.5 | 13.6 | 45.3 | 65.8 | 10.1038/mp.2015.69 |
| Queensland Twin IMaging (QTIM) | Clinical interview (CIDI) | 376 | 22.1 | 3.3 | 30.9 | 26.6 | 10.18112/openneuro.ds004169.v1.0.7 |
| Study of Health in Pomerania (SHIP-START) | Clinical interview (M-CIDI) | 562 | 55.1 | 12.5 | 48.9 | 23.3 | 10.1093/ije/dyac034 |
| SHIP-TREND (Set 1) | Clinical interview (M-CIDI) | 540 | 49.3 | 13.1 | 48.3 | 23.1 | 10.3390/healthcare10010033 |
| SHIP-TREND (Set 2) | Clinical interview (M-CIDI) | 649 | 51.1 | 14.2 | 52.1 | 26.5 | 10.3390/healthcare10010033 |
| UK Biobank (UKB) | Self-declared depressive symptoms | 40,359 | 64.1 | 7.7 | 47.4 | 16.4 | 10.1038/nn.4393 |
| Bipolar Family Study (BFS, baseline) | Clinical interview (SCID) | 144 | 23.4 | 2.8 | 47.2 | 13.2 | 10.12688/wellcomeopenres.15617.3 |
|  | **Young individuals (< 25 years old)** | | | | | | |
| **Cohort** |  | **N** | **Mean age (years)** | **SD of age** | **Male (%)** | **MD (%)** |  |
| ABCD |  | 4,826 | 9.93 | 0.63 | 52.7 | 1.8 |  |
| BFS |  | 99 | 21.90 | 1.96 | 47.5 | 10.1 |  |
| FOR2107 |  | 307 | 21.84 | 1.84 | 33.2 | 33.9 |  |
| MPIP |  | 28 | 22.13 | 1.53 | 42.9 | 67.9 |  |
| QTIM |  | 281 | 20.67 | 2.34 | 32.0 | 26.3 |  |
| SHIPTrend_batch1 |  | 13 | 23.31 | 0.95 | 46.2 | 15.4 |  |
| SHIPTrend_batch2 |  | 16 | 23.00 | 0.97 | 62.5 | 6.2 |  |

Notes: KSADS = Kiddie Schedule for Affective Disorders and Schizophrenia^10, 11^, CIDI = Composite International Diagnostic Interview^12^, and SCID = The Structured Clinical Interview for DSM^13^.

Supplementary Table 2. Correlation between PRS and PRS residuals for each individual study. PRS residuals were derived by residualizing PRS against technical covariates and measures of relatedness (GRM/kinship matrices/family structure). Correlation analysis was performed per study.

| **Study** | **PRS-CT: p = 0.01** | **PRS-CT: p = 1** | **PRS-SBayesR** |
| --- | --- | --- | --- |
| ABCD | 0.919 | 0.903 | 0.902 |
| BFS | 0.978 | 0.961 | 0.975 |
| BiDirect | 1 | 0.999 | 0.999 |
| CLING_prs1 | 1 | 1 | 1 |
| CLING_prs2 | 0.987 | 0.994 | 1 |
| CSAN | 0.999 | 1 | 0.999 |
| FOR2107 | 1 | 1 | 1 |
| GS (GS:STRADL) | 1 | 0.999 | 0.998 |
| MPIP | 1 | 1 | 1 |
| NESDA | 1 | 1 | 1 |
| QTIM | 0.99 | 0.92 | 0.983 |
| SHIP | 1 | 1 | 1 |
| SHIPTrend_batch1 | 1 | 1 | 0.999 |
| SHIPTrend_batch2 | 1 | 1 | 1 |
| UKB | 0.996 | 0.996 | 0.998 |

##### Supplementary Table 3. Prediction of MD in the testing sample.

| **PRS** | **Log(OR)** | **SE** | **p** |
| --- | --- | --- | --- |
| SBayesR | 0.181 | 0.013 | 2.83e-42 |
| CT.pT=1 | 0.162 | 0.013 | 1.70e-34 |
| CT.pT=0.5 | 0.162 | 0.013 | 1.50e-34 |
| CT.pT=0.1 | 0.133 | 0.013 | 7.23e-24 |
| CT.pT=0.01 | 0.073 | 0.013 | 3.39e-08 |
| CT.pT=1e-3 | 0.041 | 0.013 | 0.002 |
| CT.pT=1e-4 | 0.024 | 0.013 | 0.07 |
| CT.pT=5e-8 | 0.007 | 0.013 | 0.585 |

##### Supplementary Table 4. Association between global brain structural measures and all PRS in the full sample and adolescent sample. CT = PRS created using clumping and thresholding method. pT = p threshold used for creating PRS. SBayesR = PRS created using SBayesR. ICV = intracranial volume.

|  |  | Full sample | | | | Adolescent sample (age < 25 yrs) | | | |
| --- | --- | --- | --- | --- | --- | --- | --- | --- | --- |
| Brain structural measure | PRS | Beta | std | p | p.FDR | Beta | std | p | p.FDR |
| ICV | SBayesR | -0.007 | 0.004 | 0.052 | 0.078 | -0.007 | 0.012 | 0.587 | 0.922 |
| Global thickness | SBayesR | 0.001 | 0.003 | 0.639 | 0.703 | 0.002 | 0.012 | 0.874 | 0.93 |
| Global surface area | SBayesR | -0.007 | 0.003 | 0.011 | 0.02 | -2.75e-04 | 0.012 | 0.982 | 0.982 |
| ICV | CT.pT=1 | -0.017 | 0.004 | 1.97e-06 | 3.54e-05 | -0.022 | 0.012 | 0.074 | 0.422 |
| Global thickness | CT.pT=1 | 0.002 | 0.003 | 0.48 | 0.621 | -0.004 | 0.012 | 0.772 | 0.93 |
| Global surface area | CT.pT=1 | -0.013 | 0.003 | 4.50e-06 | 3.62e-05 | -0.011 | 0.012 | 0.375 | 0.806 |
| ICV | CT.pT=0.5 | -0.017 | 0.004 | 2.15e-06 | 3.54e-05 | -0.022 | 0.012 | 0.071 | 0.422 |
| Global thickness | CT.pT=0.5 | 0.002 | 0.003 | 0.491 | 0.621 | -0.004 | 0.012 | 0.762 | 0.93 |
| Global surface area | CT.pT=0.5 | -0.012 | 0.003 | 5.49e-06 | 3.62e-05 | -0.011 | 0.012 | 0.378 | 0.806 |
| ICV | CT.pT=0.1 | -0.015 | 0.004 | 2.91e-05 | 1.37e-04 | -0.02 | 0.012 | 0.111 | 0.422 |
| Global thickness | CT.pT=0.1 | 0.003 | 0.003 | 0.377 | 0.541 | -0.002 | 0.012 | 0.862 | 0.93 |
| Global surface area | CT.pT=0.1 | -0.011 | 0.003 | 4.56e-05 | 1.88e-04 | -0.009 | 0.012 | 0.442 | 0.806 |
| ICV | CT.pT=0.01 | -0.013 | 0.004 | 3.90e-04 | 0.001 | -0.02 | 0.012 | 0.102 | 0.422 |
| Global thickness | CT.pT=0.01 | 0.002 | 0.003 | 0.484 | 0.621 | -0.002 | 0.012 | 0.843 | 0.93 |
| Global surface area | CT.pT=0.01 | -0.008 | 0.003 | 0.002 | 0.005 | -0.009 | 0.012 | 0.459 | 0.806 |
| ICV | CT.pT=1e-3 | -0.011 | 0.004 | 0.002 | 0.005 | -0.019 | 0.012 | 0.134 | 0.441 |
| Global thickness | CT.pT=1e-3 | 0.002 | 0.003 | 0.597 | 0.68 | -0.002 | 0.012 | 0.85 | 0.93 |
| Global surface area | CT.pT=1e-3 | -0.007 | 0.003 | 0.01 | 0.02 | -0.009 | 0.012 | 0.475 | 0.806 |
| ICV | CT.pT=1e-4 | -0.011 | 0.004 | 0.002 | 0.005 | -0.02 | 0.012 | 0.115 | 0.422 |
| Global thickness | CT.pT=1e-4 | 0.001 | 0.003 | 0.712 | 0.734 | -0.001 | 0.012 | 0.93 | 0.959 |
| Global surface area | CT.pT=1e-4 | -0.007 | 0.003 | 0.014 | 0.024 | -0.009 | 0.012 | 0.471 | 0.806 |
| ICV | CT.pT=5e-8 | -0.011 | 0.004 | 0.002 | 0.005 | -0.02 | 0.012 | 0.107 | 0.422 |
| Global thickness | CT.pT=5e-8 | 0.001 | 0.003 | 0.696 | 0.734 | -0.002 | 0.012 | 0.873 | 0.93 |
| Global surface area | CT.pT=5e-8 | -0.006 | 0.003 | 0.023 | 0.036 | -0.009 | 0.012 | 0.479 | 0.806 |

##### Supplementary Table 5. Association between lobar brain structural measures, g factor of subcortical volume and PRS-CT at pT = 1. FDR-correction was applied per whole-brain set of measures. Associations that are significant after FDR-correction are highlighted in bold. CT = PRS created using clumping and thresholding method. pT = p threshold used for creating PRS. SBayesR = PRS created using SBayesR. ICV = intracranial volume.

|  |  | Full sample | | | | Adolescent sample (age < 25 yrs) | | | |
| --- | --- | --- | --- | --- | --- | --- | --- | --- | --- |
| Brain structural measure | PRS | Beta | std | p | p.FDR | Beta | std | p | p.FDR |
| Frontal thickness | CT.pT=1 | 0.005 | 0.003 | 0.149 | 0.475 | 0.001 | 0.01 | 0.916 | 0.957 |
| Parietal thickness | CT.pT=1 | 0.003 | 0.003 | 0.361 | 0.475 | 0.002 | 0.011 | 0.824 | 0.957 |
| Temporal thickness | CT.pT=1 | -0.002 | 0.003 | 0.538 | 0.538 | -0.01 | 0.01 | 0.329 | 0.957 |
| Occipital thickness | CT.pT=1 | 0.005 | 0.004 | 0.206 | 0.475 | -0.008 | 0.012 | 0.532 | 0.957 |
| Cingulate thickness | CT.pT=1 | 0.003 | 0.004 | 0.38 | 0.475 | 6.70E-04 | 0.012 | 0.957 | 0.957 |
| Frontal surface area | CT.pT=1 | **-0.011** | **0.002** | **2.85E-06** | **1.42E-05** | -0.013 | 1.10E-02 | 0.266 | 0.69 |
| Parietal surface area | CT.pT=1 | **-0.008** | **0.002** | **0.001** | **0.002** | -0.009 | 0.011 | 0.414 | 0.69 |
| Temporal surface area | CT.pT=1 | **-0.008** | **0.002** | **0.001** | **0.002** | -0.01 | 0.011 | 0.368 | 0.69 |
| Occipital surface area | CT.pT=1 | **-0.008** | **0.003** | **0.003** | **0.004** | -3.55E-04 | 0.012 | 0.976 | 0.976 |
| Cingulate surface area | CT.pT=1 | **-0.006** | **0.003** | **0.033** | **0.033** | -0.005 | 0.012 | 0.668 | 0.835 |
| g Subcortical volume | CT.pT=1 | **-0.011** | **0.003** | **4.41E-04** | **4.41E-04** | 0.004 | 0.012 | 0.751 | 0.751 |

##### Supplementary Table 6. Association between cortical thickness in individual regions and PRS-CT at pT = 1. CT = PRS created using clumping and thresholding method. pT = p threshold used for creating PRS. SBayesR = PRS created using SBayesR. ICV = intracranial volume. p.FDR (WB) = FDR-corrected p-values at the whole-brain level (main results). p.Bon (WB) = Bonferroni-corrected p-values at the whole-brain level. p.FDR (all) = FDR-corrected p-values across all measures.

| **Brain structural measure** | **Full sample** | | | | | | **Adolescent sample (age < 25 yrs)** | | | | | |
| --- | --- | --- | --- | --- | --- | --- | --- | --- | --- | --- | --- | --- |
|  | **Beta** | **std** | **p** | **p.FDR (WB)** | **p.Bon (WB)** | **p.FDR (all)** | **Beta** | **std** | **p** | **p.FDR (WB)** | **p.Bon (WB)** | **p.FDR (all)** |
| **Left bankssts** | -7.93E-04 | 0.004 | 0.839 | 0.955 | 1 | 0.894 | -0.003 | 0.012 | 0.804 | 0.971 | 1 | 0.975 |
| **Left caudal anterior cingulate** | 0.002 | 0.004 | 0.573 | 0.788 | 1 | 0.661 | -0.006 | 0.013 | 0.657 | 0.971 | 1 | 0.946 |
| **Left caudal middle frontal** | 0.005 | 0.003 | 0.158 | 0.695 | 1 | 0.308 | -0.012 | 0.012 | 0.332 | 0.971 | 1 | 0.945 |
| **Left cuneus** | -2.53E-04 | 0.004 | 0.948 | 0.956 | 1 | 0.961 | -0.015 | 0.012 | 0.221 | 0.971 | 1 | 0.945 |
| **Left entorhil** | 0.006 | 0.004 | 0.177 | 0.695 | 1 | 0.323 | 2.24E-04 | 0.013 | 0.986 | 0.986 | 1 | 0.993 |
| **Left fusiform** | 0.002 | 0.003 | 0.497 | 0.763 | 1 | 0.605 | -0.005 | 0.01 | 0.648 | 0.971 | 1 | 0.945 |
| **Left inferior parietal** | 2.34E-04 | 0.003 | 0.944 | 0.956 | 1 | 0.961 | -0.007 | 0.011 | 0.566 | 0.971 | 1 | 0.945 |
| **Left inferior temporal** | -4.67E-04 | 0.004 | 0.896 | 0.956 | 1 | 0.935 | -0.007 | 0.011 | 0.49 | 0.971 | 1 | 0.945 |
| **Left isthmus cingulate** | 0.002 | 0.004 | 0.536 | 0.778 | 1 | 0.644 | 0.005 | 0.013 | 0.722 | 0.971 | 1 | 0.973 |
| **Left lateral occipital** | 0.007 | 0.004 | 0.073 | 0.695 | 1 | 0.167 | -0.002 | 0.013 | 0.86 | 0.971 | 1 | 0.975 |
| **Left lateral orbitofrontal** | 0.002 | 0.004 | 0.559 | 0.785 | 1 | 0.658 | -0.011 | 0.011 | 0.334 | 0.971 | 1 | 0.945 |
| **Left lingual** | 0.005 | 0.004 | 0.22 | 0.695 | 1 | 0.383 | -0.003 | 0.012 | 0.832 | 0.971 | 1 | 0.975 |
| **Left medial orbitofrontal** | 0.003 | 0.004 | 0.396 | 0.695 | 1 | 0.517 | 0.005 | 0.012 | 0.689 | 0.971 | 1 | 0.958 |
| **Left middle temporal** | 0.001 | 0.004 | 0.736 | 0.867 | 1 | 0.796 | -0.009 | 0.011 | 0.427 | 0.971 | 1 | 0.945 |
| **Left parahippocampal** | -0.007 | 0.004 | 0.099 | 0.695 | 1 | 0.209 | -0.003 | 0.013 | 0.828 | 0.971 | 1 | 0.975 |
| **Left paracentral** | 3.46E-04 | 0.003 | 0.919 | 0.956 | 1 | 0.952 | -9.80E-04 | 0.011 | 0.931 | 0.986 | 1 | 0.993 |
| **Left parsopercularis** | 0.003 | 0.003 | 0.366 | 0.695 | 1 | 0.501 | -0.014 | 0.012 | 0.241 | 0.971 | 1 | 0.945 |
| **Left parsorbitalis** | 3.06E-04 | 0.004 | 0.936 | 0.956 | 1 | 0.961 | -0.007 | 0.012 | 0.531 | 0.971 | 1 | 0.945 |
| **Left parstriangularis** | 0.007 | 0.004 | 0.064 | 0.695 | 1 | 0.157 | 0.002 | 0.011 | 0.835 | 0.971 | 1 | 0.975 |
| **Left pericalcarine** | 0.003 | 0.004 | 0.387 | 0.695 | 1 | 0.508 | 0.003 | 0.013 | 0.806 | 0.971 | 1 | 0.975 |
| **Left post central** | 0.002 | 0.004 | 0.668 | 0.83 | 1 | 0.744 | -0.004 | 0.012 | 0.733 | 0.971 | 1 | 0.973 |
| **Left posterior cingulate** | 0.004 | 0.004 | 0.296 | 0.695 | 1 | 0.45 | 0.004 | 0.013 | 0.774 | 0.971 | 1 | 0.975 |
| **Left precentral** | 0.003 | 0.003 | 0.354 | 0.695 | 1 | 0.497 | -0.008 | 0.013 | 0.539 | 0.971 | 1 | 0.945 |
| **Left precuneus** | 0.003 | 0.003 | 0.33 | 0.695 | 1 | 0.478 | 2.11E-04 | 0.011 | 0.985 | 0.986 | 1 | 0.993 |
| **Left rostral anterior cingulate** | -6.52E-04 | 0.004 | 0.871 | 0.956 | 1 | 0.921 | -0.022 | 0.013 | 0.086 | 0.971 | 1 | 0.945 |
| **Left rostral middle frontal** | 0.003 | 0.003 | 0.315 | 0.695 | 1 | 0.468 | -0.011 | 0.011 | 0.346 | 0.971 | 1 | 0.945 |
| **Left superior frontal** | 0.001 | 0.003 | 0.688 | 0.83 | 1 | 0.754 | -0.004 | 0.012 | 0.75 | 0.971 | 1 | 0.975 |
| **Left superior parietal** | 0.003 | 0.003 | 0.432 | 0.695 | 1 | 0.539 | 0.006 | 0.012 | 0.586 | 0.971 | 1 | 0.945 |
| **Left superior temporal** | -0.004 | 0.004 | 0.274 | 0.695 | 1 | 0.426 | 0.001 | 0.012 | 0.908 | 0.986 | 1 | 0.993 |
| **Left supramargil** | 0.002 | 0.003 | 0.472 | 0.742 | 1 | 0.579 | 0.006 | 0.012 | 0.628 | 0.971 | 1 | 0.945 |
| **Left frontal pole** | 0.003 | 0.004 | 0.428 | 0.695 | 1 | 0.539 | 0.017 | 0.012 | 0.177 | 0.971 | 1 | 0.945 |
| **Left transverse temporal** | 0.004 | 0.004 | 0.352 | 0.695 | 1 | 0.497 | 0.012 | 0.011 | 0.28 | 0.971 | 1 | 0.945 |
| **Left insula** | -0.008 | 0.004 | 0.035 | 0.695 | 1 | 0.092 | -0.017 | 0.012 | 0.151 | 0.971 | 1 | 0.945 |
| **Right bankssts** | -0.006 | 0.004 | 0.147 | 0.695 | 1 | 0.29 | -0.012 | 0.012 | 0.3 | 0.971 | 1 | 0.945 |
| **Right caudal anterior cingulate** | 0.004 | 0.004 | 0.317 | 0.695 | 1 | 0.468 | -0.008 | 0.013 | 0.563 | 0.971 | 1 | 0.945 |
| **Right caudal middle frontal** | 0.001 | 0.004 | 0.753 | 0.872 | 1 | 0.808 | -0.008 | 0.012 | 0.49 | 0.971 | 1 | 0.945 |
| **Right cuneus** | 0.003 | 0.004 | 0.38 | 0.695 | 1 | 0.504 | -0.016 | 0.013 | 0.201 | 0.971 | 1 | 0.945 |
| **Right entorhil** | -0.002 | 0.004 | 0.587 | 0.791 | 1 | 0.67 | -0.005 | 0.013 | 0.667 | 0.971 | 1 | 0.946 |
| **Right fusiform** | -0.001 | 0.004 | 0.692 | 0.83 | 1 | 0.754 | -0.012 | 0.011 | 0.279 | 0.971 | 1 | 0.945 |
| **Right inferior parietal** | 0.001 | 0.003 | 0.691 | 0.83 | 1 | 0.754 | 0.008 | 0.012 | 0.474 | 0.971 | 1 | 0.945 |
| **Right inferior temporal** | 0.003 | 0.004 | 0.367 | 0.695 | 1 | 0.501 | -0.009 | 0.011 | 0.395 | 0.971 | 1 | 0.945 |
| **Right isthmus cingulate** | -0.002 | 0.004 | 0.542 | 0.778 | 1 | 0.644 | 0.017 | 0.013 | 0.211 | 0.971 | 1 | 0.945 |
| **Right lateral occipital** | 0.006 | 0.004 | 0.092 | 0.695 | 1 | 0.2 | -8.76E-04 | 0.013 | 0.946 | 0.986 | 1 | 0.993 |
| **Right lateral orbitofrontal** | 0.004 | 0.004 | 0.24 | 0.695 | 1 | 0.405 | -0.006 | 0.011 | 0.558 | 0.971 | 1 | 0.945 |
| **Right lingual** | 0.003 | 0.004 | 0.419 | 0.695 | 1 | 0.539 | -0.017 | 0.012 | 0.159 | 0.971 | 1 | 0.945 |
| **Right medial orbito-frontal** | 0.006 | 0.004 | 0.1 | 0.695 | 1 | 0.209 | 0.002 | 0.011 | 0.834 | 0.971 | 1 | 0.975 |
| **Right middle temporal** | -0.005 | 0.004 | 0.205 | 0.695 | 1 | 0.37 | -0.007 | 0.011 | 0.514 | 0.971 | 1 | 0.945 |
| **Right parahippocampal** | 0.005 | 0.004 | 0.259 | 0.695 | 1 | 0.417 | 0.007 | 0.012 | 0.58 | 0.971 | 1 | 0.945 |
| **Right paracentral** | 0.004 | 0.003 | 0.22 | 0.695 | 1 | 0.383 | 0.005 | 0.011 | 0.631 | 0.971 | 1 | 0.945 |
| **Right parsopercularis** | 0.002 | 0.004 | 0.539 | 0.778 | 1 | 0.644 | -2.38E-04 | 0.012 | 0.984 | 0.986 | 1 | 0.993 |
| **Right parsorbitalis** | 0.006 | 0.004 | 0.098 | 0.695 | 1 | 0.209 | 0.008 | 0.011 | 0.481 | 0.971 | 1 | 0.945 |
| **Right parstriangularis** | 0.006 | 0.004 | 0.115 | 0.695 | 1 | 0.23 | 0.012 | 0.011 | 0.28 | 0.971 | 1 | 0.945 |
| **Right pericalcarine** | 5.25E-04 | 0.004 | 0.897 | 0.956 | 1 | 0.935 | -0.005 | 0.013 | 0.708 | 0.971 | 1 | 0.973 |
| **Right post central** | 0.004 | 0.004 | 0.288 | 0.695 | 1 | 0.442 | 4.94E-04 | 0.012 | 0.968 | 0.986 | 1 | 0.993 |
| **Right posterior cingulate** | 0.002 | 0.004 | 0.667 | 0.83 | 1 | 0.744 | 0.037 | 0.013 | 0.004 | 0.271 | 0.271 | 0.6 |
| **Right precentral** | -1.96E-04 | 0.004 | 0.956 | 0.956 | 1 | 0.963 | -0.007 | 0.012 | 0.578 | 0.971 | 1 | 0.945 |
| **Right precuneus** | 0.005 | 0.003 | 0.112 | 0.695 | 1 | 0.23 | 0.007 | 0.011 | 0.527 | 0.971 | 1 | 0.945 |
| **Right rostral anterior cingulate** | 0.004 | 0.004 | 0.307 | 0.695 | 1 | 0.462 | -0.01 | 0.013 | 0.418 | 0.971 | 1 | 0.945 |
| **Right rostral middle frontal** | 0.008 | 0.003 | 0.023 | 0.695 | 1 | 0.069 | 0.007 | 0.01 | 0.502 | 0.971 | 1 | 0.945 |
| **Right superior frontal** | 0.003 | 0.003 | 0.344 | 0.695 | 1 | 0.492 | -0.002 | 0.011 | 0.856 | 0.971 | 1 | 0.975 |
| **Right superior parietal** | 0.003 | 0.003 | 0.379 | 0.695 | 1 | 0.504 | -0.003 | 0.012 | 0.821 | 0.971 | 1 | 0.975 |
| **Right superior temporal** | -0.004 | 0.004 | 0.273 | 0.695 | 1 | 0.426 | -0.006 | 0.011 | 0.625 | 0.971 | 1 | 0.945 |
| **Right supramargil** | 0.003 | 0.003 | 0.431 | 0.695 | 1 | 0.539 | 0.002 | 0.012 | 0.868 | 0.971 | 1 | 0.975 |
| **Right frontal pole** | 0.005 | 0.004 | 0.173 | 0.695 | 1 | 0.323 | 0.017 | 0.012 | 0.153 | 0.971 | 1 | 0.945 |
| **Right transverse temporal** | 0.002 | 0.004 | 0.632 | 0.83 | 1 | 0.715 | 0.005 | 0.012 | 0.646 | 0.971 | 1 | 0.945 |
| **Right insula** | -0.004 | 0.004 | 0.251 | 0.695 | 1 | 0.416 | -0.018 | 0.012 | 0.124 | 0.971 | 1 | 0.945 |

##### Supplementary Table 7. Association between cortical surface area in individual regions and PRS-CT at pT = 1. CT = PRS created using clumping and thresholding method. pT = p threshold used for creating PRS. SBayesR = PRS created using SBayesR. ICV = intracranial volume. p.FDR (WB) = FDR-corrected p-values at the whole-brain level (main results). p.Bon (WB) = Bonferroni-corrected p-values at the whole-brain level. p.FDR (all) = FDR-corrected p-values across all measures.

| **Brain structural measure** | **Full sample** | | | | | | **Adolescent sample (age < 25 yrs)** | | | | | |
| --- | --- | --- | --- | --- | --- | --- | --- | --- | --- | --- | --- | --- |
|  | **Beta** | **std** | **p** | **p.FDR (WB)** | **p.Bon (WB)** | **p.FDR (all)** | **Beta** | **std** | **p** | **p.FDR (WB)** | **p.Bon (WB)** | **p.FDR (all)** |
| **Left bankssts** | -9.06E-05 | 0.004 | 0.981 | 0.981 | 1 | 0.981 | -0.006 | 0.013 | 0.64 | 0.927 | 1 | 0.945 |
| **Left caudal anterior cingulate** | -0.003 | 0.004 | 0.448 | 0.469 | 1 | 0.554 | 0.003 | 0.013 | 0.789 | 0.955 | 1 | 0.975 |
| **Left caudal middle frontal** | -0.009 | 0.004 | 0.024 | 0.037 | 1 | 0.069 | -0.004 | 0.013 | 0.776 | 0.955 | 1 | 0.975 |
| **Left cuneus** | -0.005 | 0.004 | 0.242 | 0.275 | 1 | 0.405 | -0.001 | 0.013 | 0.919 | 0.987 | 1 | 0.993 |
| **Left entorhil** | -0.017 | 0.004 | 3.38E-05 | 5.57e-04 | 0.002 | 9.68E-04 | -0.008 | 0.013 | 0.514 | 0.927 | 1 | 0.945 |
| **Left fusiform** | -0.012 | 0.004 | 9.00E-04 | 0.003 | 0.059 | 0.005 | -0.012 | 0.012 | 0.342 | 0.927 | 1 | 0.945 |
| **Left inferior parietal** | -0.007 | 0.004 | 0.08 | 0.103 | 1 | 0.176 | -0.006 | 0.012 | 0.595 | 0.927 | 1 | 0.945 |
| **Left inferior temporal** | -0.015 | 0.004 | 7.17E-05 | 6.64e-04 | 0.005 | 0.001 | -0.009 | 0.012 | 0.454 | 0.927 | 1 | 0.945 |
| **Left isthmus cingulate** | -0.011 | 0.004 | 0.003 | 0.007 | 0.222 | 0.014 | -0.029 | 0.012 | 0.019 | 0.747 | 1 | 0.826 |
| **Left lateral occipital** | -0.013 | 0.004 | 7.54E-04 | 0.002 | 0.05 | 0.005 | -0.011 | 0.012 | 0.376 | 0.927 | 1 | 0.945 |
| **Left lateral orbitofrontal** | -0.015 | 0.004 | 9.19E-05 | 6.64e-04 | 0.006 | 0.001 | 5.18E-05 | 0.012 | 0.997 | 0.997 | 1 | 0.997 |
| **Left lingual** | -0.007 | 0.004 | 0.072 | 0.099 | 1 | 0.167 | 0.008 | 0.013 | 0.511 | 0.927 | 1 | 0.945 |
| **Left medial orbitofrontal** | -0.021 | 0.004 | 9.48E-08 | 6.26e-06 | 6.26e-06 | 1.38E-05 | -0.01 | 0.013 | 0.411 | 0.927 | 1 | 0.945 |
| **Left middle temporal** | -0.012 | 0.004 | 0.001 | 0.003 | 0.074 | 0.006 | -0.015 | 0.012 | 0.213 | 0.927 | 1 | 0.945 |
| **Left parahippocampal** | -0.004 | 0.004 | 0.327 | 0.354 | 1 | 0.477 | 0.005 | 0.013 | 0.716 | 0.955 | 1 | 0.973 |
| **Left paracentral** | -0.011 | 0.004 | 0.008 | 0.014 | 0.515 | 0.027 | -0.013 | 0.013 | 0.321 | 0.927 | 1 | 0.945 |
| **Left parsopercularis** | -0.011 | 0.004 | 0.005 | 0.011 | 0.361 | 0.021 | 0.02 | 0.013 | 0.123 | 0.927 | 1 | 0.945 |
| **Left parsorbitalis** | -0.014 | 0.004 | 3.86E-04 | 0.001 | 0.025 | 0.003 | -0.008 | 0.012 | 0.505 | 0.927 | 1 | 0.945 |
| **Left parstriangularis** | -0.01 | 0.004 | 0.015 | 0.025 | 1 | 0.05 | 0.016 | 0.013 | 0.211 | 0.927 | 1 | 0.945 |
| **Left pericalcarine** | -0.007 | 0.004 | 0.115 | 0.146 | 1 | 0.23 | 0.009 | 0.013 | 0.498 | 0.927 | 1 | 0.945 |
| **Left post central** | -0.01 | 0.004 | 0.007 | 0.013 | 0.475 | 0.026 | -0.021 | 0.012 | 0.079 | 0.927 | 1 | 0.945 |
| **Left posterior cingulate** | -0.005 | 0.004 | 0.169 | 0.207 | 1 | 0.321 | -0.01 | 0.013 | 0.402 | 0.927 | 1 | 0.945 |
| **Left precentral** | -0.015 | 0.004 | 1.04E-04 | 6.64e-04 | 0.007 | 0.001 | -0.028 | 0.012 | 0.023 | 0.747 | 1 | 0.826 |
| **Left precuneus** | -0.012 | 0.004 | 0.002 | 0.004 | 0.104 | 0.008 | -0.011 | 0.012 | 0.376 | 0.927 | 1 | 0.945 |
| **Left rostral anterior cingulate** | -0.005 | 0.004 | 0.24 | 0.275 | 1 | 0.405 | 0.017 | 0.013 | 0.178 | 0.927 | 1 | 0.945 |
| **Left rostral middle frontal** | -0.018 | 0.004 | 6.88E-07 | 2.27e-05 | 4.54e-05 | 5.02E-05 | -0.023 | 0.012 | 0.051 | 0.927 | 1 | 0.945 |
| **Left superior frontal** | -0.016 | 0.004 | 2.40E-05 | 5.29e-04 | 0.002 | 8.77E-04 | -0.016 | 0.012 | 0.186 | 0.927 | 1 | 0.945 |
| **Left superior parietal** | -0.011 | 0.004 | 0.004 | 0.009 | 0.274 | 0.017 | 0.001 | 0.012 | 0.93 | 0.987 | 1 | 0.993 |
| **Left superior temporal** | -0.01 | 0.004 | 0.008 | 0.014 | 0.53 | 0.027 | -0.013 | 0.012 | 0.291 | 0.927 | 1 | 0.945 |
| **Left supramargil** | -0.005 | 0.004 | 0.165 | 0.206 | 1 | 0.318 | 0.007 | 0.012 | 0.568 | 0.927 | 1 | 0.945 |
| **Left frontal pole** | -0.013 | 0.004 | 5.46E-04 | 0.002 | 0.036 | 0.004 | -0.009 | 0.013 | 0.473 | 0.927 | 1 | 0.945 |
| **Left transverse temporal** | -0.005 | 0.004 | 0.274 | 0.302 | 1 | 0.426 | -0.016 | 0.013 | 0.204 | 0.927 | 1 | 0.945 |
| **Left insula** | -0.007 | 0.004 | 0.073 | 0.099 | 1 | 0.167 | 0.013 | 0.012 | 0.309 | 0.927 | 1 | 0.945 |
| **Right bankssts** | -0.009 | 0.004 | 0.016 | 0.026 | 1 | 0.051 | -0.01 | 0.012 | 0.413 | 0.927 | 1 | 0.945 |
| **Right caudal anterior cingulate** | -0.005 | 0.004 | 0.177 | 0.212 | 1 | 0.323 | 0.006 | 0.013 | 0.64 | 0.927 | 1 | 0.945 |
| **Right caudal middle frontal** | -0.012 | 0.004 | 0.002 | 0.006 | 0.159 | 0.011 | -0.002 | 0.013 | 0.869 | 0.955 | 1 | 0.975 |
| **Right cuneus** | -0.002 | 0.004 | 0.568 | 0.584 | 1 | 0.661 | 0.004 | 0.013 | 0.751 | 0.955 | 1 | 0.975 |
| **Right entorhil** | -0.015 | 0.004 | 3.52E-04 | 0.001 | 0.023 | 0.003 | -0.034 | 0.013 | 0.01 | 0.637 | 1 | 0.705 |
| **Right fusiform** | -0.014 | 0.004 | 1.21E-04 | 6.64e-04 | 0.008 | 0.001 | -0.01 | 0.012 | 0.416 | 0.927 | 1 | 0.945 |
| **Right inferior parietal** | -0.012 | 0.004 | 0.001 | 0.003 | 0.067 | 0.006 | -0.014 | 0.012 | 0.237 | 0.927 | 1 | 0.945 |
| **Right inferior temporal** | -0.013 | 0.004 | 7.18E-04 | 0.002 | 0.047 | 0.005 | -0.019 | 0.012 | 0.119 | 0.927 | 1 | 0.945 |
| **Right isthmus cingulate** | -0.007 | 0.004 | 0.078 | 0.103 | 1 | 0.175 | -0.022 | 0.013 | 0.087 | 0.927 | 1 | 0.945 |
| **Right lateral occipital** | -0.015 | 0.004 | 1.12E-04 | 6.64e-04 | 0.007 | 0.001 | -0.01 | 0.012 | 0.398 | 0.927 | 1 | 0.945 |
| **Right lateral orbitofrontal** | -0.014 | 0.004 | 2.37E-04 | 0.001 | 0.016 | 0.002 | 0.01 | 0.012 | 0.405 | 0.927 | 1 | 0.945 |
| **Right lingual** | -0.009 | 0.004 | 0.031 | 0.044 | 1 | 0.084 | 0.003 | 0.013 | 0.812 | 0.955 | 1 | 0.975 |
| **Right medial orbito-frontal** | -0.015 | 0.004 | 5.19E-05 | 5.83e-04 | 0.003 | 9.68E-04 | -0.013 | 0.013 | 0.294 | 0.927 | 1 | 0.945 |
| **Right middle temporal** | -0.007 | 0.004 | 0.057 | 0.08 | 1 | 0.141 | -0.006 | 0.012 | 0.6 | 0.927 | 1 | 0.945 |
| **Right parahippocampal** | -0.015 | 0.004 | 1.07E-04 | 6.64e-04 | 0.007 | 0.001 | -0.012 | 0.013 | 0.355 | 0.927 | 1 | 0.945 |
| **Right paracentral** | -0.012 | 0.004 | 0.003 | 0.007 | 0.218 | 0.014 | 9.52E-04 | 0.013 | 0.941 | 0.987 | 1 | 0.993 |
| **Right parsopercularis** | -0.004 | 0.004 | 0.359 | 0.382 | 1 | 0.499 | -8.15E-04 | 0.013 | 0.949 | 0.987 | 1 | 0.993 |
| **Right parsorbitalis** | -0.014 | 0.004 | 2.98E-04 | 0.001 | 0.02 | 0.002 | -0.015 | 0.012 | 0.209 | 0.927 | 1 | 0.945 |
| **Right parstriangularis** | -0.009 | 0.004 | 0.027 | 0.04 | 1 | 0.077 | -0.006 | 0.013 | 0.62 | 0.927 | 1 | 0.945 |
| **Right pericalcarine** | -0.002 | 0.004 | 0.575 | 0.584 | 1 | 0.661 | 0.01 | 0.013 | 0.433 | 0.927 | 1 | 0.945 |
| **Right post central** | -0.004 | 0.004 | 0.26 | 0.291 | 1 | 0.417 | -0.006 | 0.012 | 0.6 | 0.927 | 1 | 0.945 |
| **Right posterior cingulate** | -0.01 | 0.004 | 0.01 | 0.018 | 0.686 | 0.035 | -0.013 | 0.012 | 0.314 | 0.927 | 1 | 0.945 |
| **Right precentral** | -0.012 | 0.004 | 0.001 | 0.003 | 0.085 | 0.007 | -0.011 | 0.012 | 0.386 | 0.927 | 1 | 0.945 |
| **Right precuneus** | -0.011 | 0.004 | 0.003 | 0.007 | 0.207 | 0.014 | -0.023 | 0.012 | 0.053 | 0.927 | 1 | 0.945 |
| **Right rostral anterior cingulate** | -0.011 | 0.004 | 0.007 | 0.013 | 0.469 | 0.026 | 0.004 | 0.013 | 0.731 | 0.955 | 1 | 0.973 |
| **Right rostral middle frontal** | -0.014 | 0.004 | 1.48E-04 | 7.53e-04 | 0.01 | 0.001 | -0.023 | 0.012 | 0.055 | 0.927 | 1 | 0.945 |
| **Right superior frontal** | -0.015 | 0.004 | 5.30E-05 | 5.83e-04 | 0.003 | 9.68E-04 | -0.011 | 0.012 | 0.356 | 0.927 | 1 | 0.945 |
| **Right superior parietal** | -0.01 | 0.004 | 0.007 | 0.013 | 0.451 | 0.026 | 0.014 | 0.012 | 0.262 | 0.927 | 1 | 0.945 |
| **Right superior temporal** | -0.013 | 0.004 | 0.001 | 0.003 | 0.067 | 0.006 | -0.005 | 0.012 | 0.665 | 0.927 | 1 | 0.946 |
| **Right supramargil** | -0.009 | 0.004 | 0.02 | 0.031 | 1 | 0.061 | -0.002 | 0.012 | 0.867 | 0.955 | 1 | 0.975 |
| **Right frontal pole** | -0.009 | 0.004 | 0.026 | 0.039 | 1 | 0.074 | -0.024 | 0.013 | 0.064 | 0.927 | 1 | 0.945 |
| **Right transverse temporal** | -0.005 | 0.004 | 0.221 | 0.26 | 1 | 0.383 | -0.003 | 0.013 | 0.811 | 0.955 | 1 | 0.975 |
| **Right insula** | -0.011 | 0.004 | 0.005 | 0.01 | 0.323 | 0.019 | -0.011 | 0.012 | 0.369 | 0.927 | 1 | 0.945 |

##### Supplementary Table 8. Association between subcortical volume in individual regions and PRS-CT at pT = 1. CT = PRS created using clumping and thresholding method. pT = p threshold used for creating PRS. SBayesR = PRS created using SBayesR. ICV = intracranial volume. p.FDR (WB) = FDR-corrected p-values at the whole-brain level (main results). p.Bon (WB) = Bonferroni-corrected p-values at the whole-brain level. p.FDR (all) = FDR-corrected p-values across all measures.

| **Brain structural measure** | **Full sample** | | | | | | **Adolescent sample (age < 25 yrs)** | | | | | |
| --- | --- | --- | --- | --- | --- | --- | --- | --- | --- | --- | --- | --- |
|  | **Beta** | **std** | **p** | **p.FDR (WB)** | **p.Bon (WB)** | **p.FDR (all)** | **Beta** | **std** | **p** | **p.FDR (WB)** | **p.Bon (WB)** | **p.FDR (all)** |
| Left thalamus | -0.013 | 0.003 | 1.64E-04 | 7.65E-04 | 0.002 | 0.001 | -0.01 | 0.013 | 0.439 | 0.937 | 1 | 0.945 |
| Right thalamus | -0.015 | 0.004 | 1.73E-05 | 2.42E-04 | 2.42E-04 | 8.41E-04 | -0.008 | 0.012 | 0.535 | 0.937 | 1 | 0.945 |
| Left caudate | -0.003 | 0.004 | 0.376 | 0.405 | 1 | 0.504 | 0.013 | 0.013 | 0.328 | 0.937 | 1 | 0.945 |
| Right caudate | -0.004 | 0.004 | 0.257 | 0.3 | 1 | 0.417 | 0.009 | 0.013 | 0.474 | 0.937 | 1 | 0.945 |
| Left putamen | -0.006 | 0.003 | 0.068 | 0.086 | 0.947 | 0.162 | 0.009 | 0.013 | 0.496 | 0.937 | 1 | 0.945 |
| Right putamen | -0.003 | 0.003 | 0.432 | 0.432 | 1 | 0.539 | 0.022 | 0.012 | 0.083 | 0.937 | 1 | 0.945 |
| Left pallidum | -0.011 | 0.003 | 0.001 | 0.004 | 0.015 | 0.006 | 0.002 | 0.012 | 0.865 | 0.986 | 1 | 0.975 |
| Right pallidum | -0.014 | 0.003 | 4.19E-05 | 2.94E-04 | 5.87E-04 | 9.68E-04 | -0.005 | 0.013 | 0.679 | 0.986 | 1 | 0.953 |
| Left hippocampus | -0.012 | 0.004 | 0.002 | 0.006 | 0.03 | 0.01 | 2.30E-04 | 0.013 | 0.986 | 0.986 | 1 | 0.993 |
| Right hippocampus | -0.008 | 0.004 | 0.032 | 0.057 | 0.452 | 0.086 | -0.008 | 0.013 | 0.514 | 0.937 | 1 | 0.945 |
| Left amygdala | -0.008 | 0.004 | 0.028 | 0.057 | 0.397 | 0.078 | -0.013 | 0.013 | 0.306 | 0.937 | 1 | 0.945 |
| Right amygdala | -0.007 | 0.004 | 0.051 | 0.072 | 0.72 | 0.129 | -9.22E-04 | 0.013 | 0.941 | 0.986 | 1 | 0.993 |
| Left accumbens | -0.007 | 0.003 | 0.043 | 0.066 | 0.598 | 0.109 | 0.001 | 0.013 | 0.926 | 0.986 | 1 | 0.993 |
| Right accumbens | -0.008 | 0.003 | 0.023 | 0.053 | 0.317 | 0.069 | 6.81E-04 | 0.013 | 0.958 | 0.986 | 1 | 0.993 |

##### Supplementary Table 9. Instrumental variants used in the Mendelian randomisation analysis of causal effect from left hippocampal volume to liability of MD. Statistics for exposure (left hippocampal volume) GWAS were annotated with the suffix ‘.exposure’, and statistics for outcome (liability of MD) GWAS were annotated with the suffix ‘.outcome’. The column ‘eaf’: allele frequency for effect allele (A1).

|  |  |  |  |  | Exposure | | | | Outcome | | | |
| --- | --- | --- | --- | --- | --- | --- | --- | --- | --- | --- | --- | --- |
| SNP | CHR | BP | A1 | A2 | Beta | SE | p | Freq | Beta | SE | p | Freq |
| rs12218394 | 10 | 126480591 | C | G | -0.069 | 0.008 | 3.50e-18 | 0.57 | 0.005 | 0.004 | 0.167 | 0.573 |
| rs12817240 | 12 | 41846898 | C | T | -0.082 | 0.015 | 2.89e-08 | 0.08 | 0.017 | 0.006 | 0.01 | 0.075 |
| rs146607495 | 12 | 117319202 | T | C | 0.181 | 0.014 | 5.11e-41 | 0.096 | -0.004 | 0.006 | 0.472 | 0.099 |
| rs17178006 | 12 | 65718299 | G | T | -0.137 | 0.012 | 2.43e-29 | 0.116 | 0.008 | 0.006 | 0.164 | 0.107 |
| rs2578475 | 12 | 4007324 | G | T | -0.049 | 0.008 | 4.89e-09 | 0.344 | -0.004 | 0.004 | 0.25 | 0.34 |
| rs28740897 | 6 | 150108141 | A | G | 0.055 | 0.008 | 6.74e-11 | 0.339 | 0.008 | 0.005 | 0.121 | 0.338 |
| rs28758826 | 15 | 98443571 | G | A | -0.047 | 0.008 | 3.78e-09 | 0.548 | 0.008 | 0.004 | 0.026 | 0.547 |
| rs2970931 | 2 | 162873188 | C | T | -0.071 | 0.008 | 8.63e-19 | 0.57 | 0.005 | 0.004 | 0.188 | 0.584 |
| rs3852188 | 5 | 82858014 | G | C | 0.067 | 0.01 | 2.58e-11 | 0.194 | -1.00e-03 | 0.004 | 0.818 | 0.194 |
| rs4947122 | 6 | 111870090 | T | C | -0.055 | 0.01 | 3.62e-08 | 0.202 | 0.021 | 0.004 | 1.18e-06 | 0.209 |
| rs61192764 | 6 | 108995187 | G | A | -0.074 | 0.012 | 1.21e-09 | 0.123 | 0.011 | 0.005 | 0.035 | 0.121 |
| rs61785580 | 1 | 46402849 | T | C | -0.099 | 0.015 | 1.46e-10 | 0.072 | 0.004 | 0.007 | 0.516 | 0.074 |
| rs6699195 | 1 | 54841838 | C | T | -0.065 | 0.008 | 1.21e-16 | 0.501 | 0.005 | 0.004 | 0.156 | 0.503 |
| rs6975613 | 7 | 148868109 | G | T | -0.053 | 0.008 | 3.30e-10 | 0.618 | 0.005 | 0.004 | 0.178 | 0.548 |
| rs7030607 | 9 | 119245183 | A | G | 0.079 | 0.008 | 1.47e-21 | 0.36 | -8.00e-04 | 0.004 | 0.82 | 0.354 |
| rs9829866 | 3 | 171092891 | T | A | 0.047 | 0.009 | 4.20e-08 | 0.306 | -0.007 | 0.004 | 0.079 | 0.304 |

##### Supplementary Table 10. Validation analysis of causal effect from left hippocampal volume to liability of MD. Sensitivity analysis was conducted after removing rs4947122, rs28758826 and rs61192764 which showed medium level LD (r>0.15) with other IVs in the post-hoc quality check (Supplementary Figure 4-6).

| Analysis | Exposure | Outcome | MR method | Nsnp | Beta | SE | p | Egger intercept | Egger p-value | Q statistics | Q p-value |
| --- | --- | --- | --- | --- | --- | --- | --- | --- | --- | --- | --- |
| Main analysis | Left hippocampus | MDD | Inverse variance weighted | 15 | -0.064 | 0.024 | 0.008 | -0.005 | 0.346 | 36.89 | 7.67e-04 |
| Main analysis | Left hippocampus | MDD | MR Egger (bootstrap) | 15 | -6.33e-04 | 0.053 | 0.514 | -0.005 | 0.346 | 36.89 | 7.67e-04 |
| Main analysis | Left hippocampus | MDD | Weighted median | 15 | -0.049 | 0.021 | 0.019 | -0.005 | 0.346 | 36.89 | 7.67e-04 |
| Validation analysis | Left hippocampus | MDD | Inverse variance weighted | 12 | -0.042 | 0.019 | 0.024 | 3.65e-05 | 0.993 | 14.985 | 0.183 |
| Validation analysis | Left hippocampus | MDD | MR Egger (bootstrap) | 12 | -0.034 | 0.051 | 0.255 | 3.65e-05 | 0.993 | 14.985 | 0.183 |
| Validation analysis | Left hippocampus | MDD | Weighted median | 12 | -0.041 | 0.02 | 0.044 | 3.65e-05 | 0.993 | 14.985 | 0.183 |

Supplementary Table 11. Interaction of sex and PRS-CT at p=1 for MD on global structural measures.

| **Global structural measure** | **Beta** | **std** | **t-value** | **p-value** | **pFDR** |
| --- | --- | --- | --- | --- | --- |
| Mean thickness | 0.007 | 0.006 | 1.090 | 0.276 | 0.413 |
| Total surface area | 0.003 | 0.005 | 0.563 | 0.573 | 0.573 |
| ICV | 0.010 | 0.007 | 1.420 | 0.155 | 0.413 |

Supplementary Table 12. Interaction of sex and PRS-CT at p=1 for MD on global structural measures.

|  | **Lobar structural measure** | **Beta** | **std** | **t-value** | **p-value** | **pFDR** |
| --- | --- | --- | --- | --- | --- | --- |
| Cortical thickness | Frontal | 0.006 | 0.006 | 0.931 | 0.352 | 0.663 |
|  | Parietal | 0.006 | 0.006 | 0.939 | 0.348 | 0.663 |
|  | Temporal | 0.004 | 0.007 | 0.600 | 0.548 | 0.783 |
|  | Occipital | 0.014 | 0.007 | 1.847 | 0.065 | 0.647 |
|  | Cingulate | 0.003 | 0.008 | 0.443 | 0.658 | 0.804 |
| Cortical surface area | Frontal | 0.005 | 0.005 | 0.981 | 0.326 | 0.663 |
|  | Parietal | -0.004 | 0.005 | -0.845 | 0.398 | 0.663 |
|  | Temporal | -0.001 | 0.005 | -0.206 | 0.836 | 0.836 |
|  | Occipital | 0.006 | 0.006 | 1.101 | 0.271 | 0.663 |
|  | Cingulate | -0.002 | 0.005 | -0.353 | 0.724 | 0.804 |

##### Supplementary Figure 1. Scree plot for PCA analysis on subcortical volumes and loadings for the first unrotated principal component (PC). Panel (a) shows the variance explained (R2, %) by each individual PC. Panel (b) shows the loadings of individual subcortical volumes on the first unrotated PC.


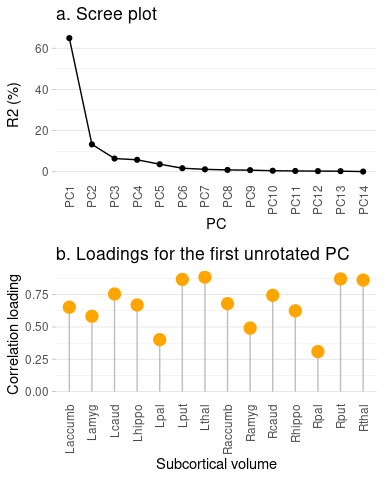


Supplementary Figure 2. Correlation between PRS and PRS residuals for PRC-CT at p=1. PRS residuals were derived by residualizing PRS against technical covariates and measures of relatedness (GRM/kinship matrices/family structure). Correlation analysis was performed and plotted per study. GS_stradl = Generation Scotland.

**
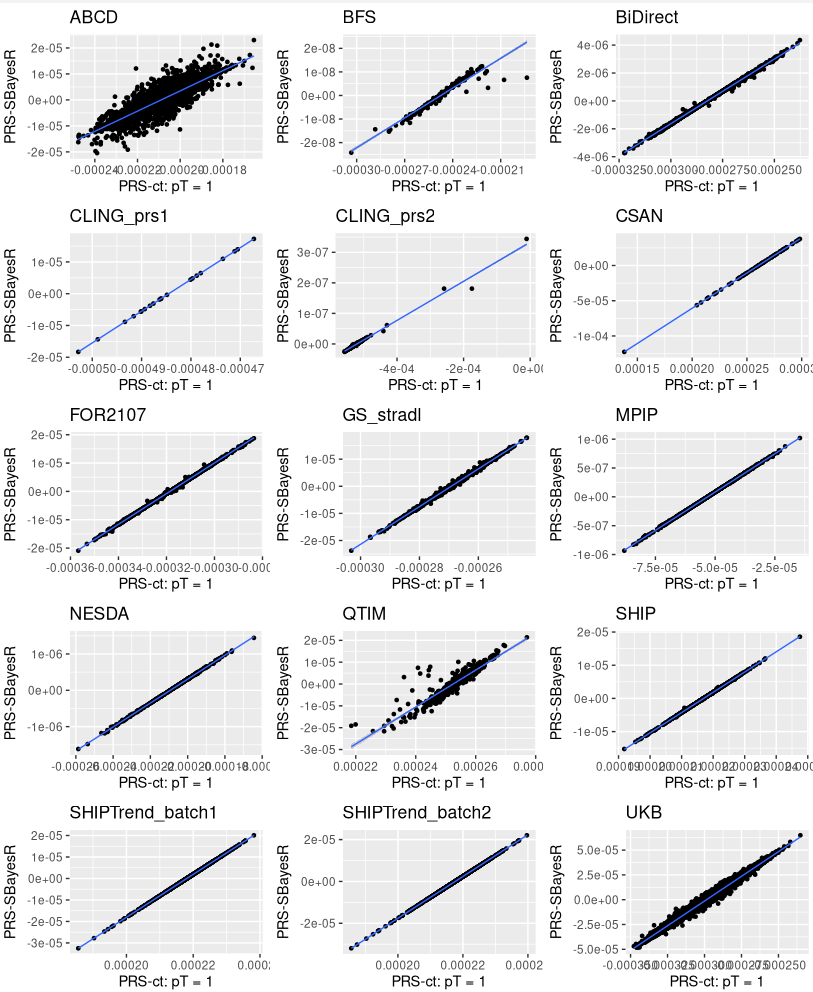
**

##### Supplementary Figure 3. PRS prediction of life-time MD in individual cohorts.


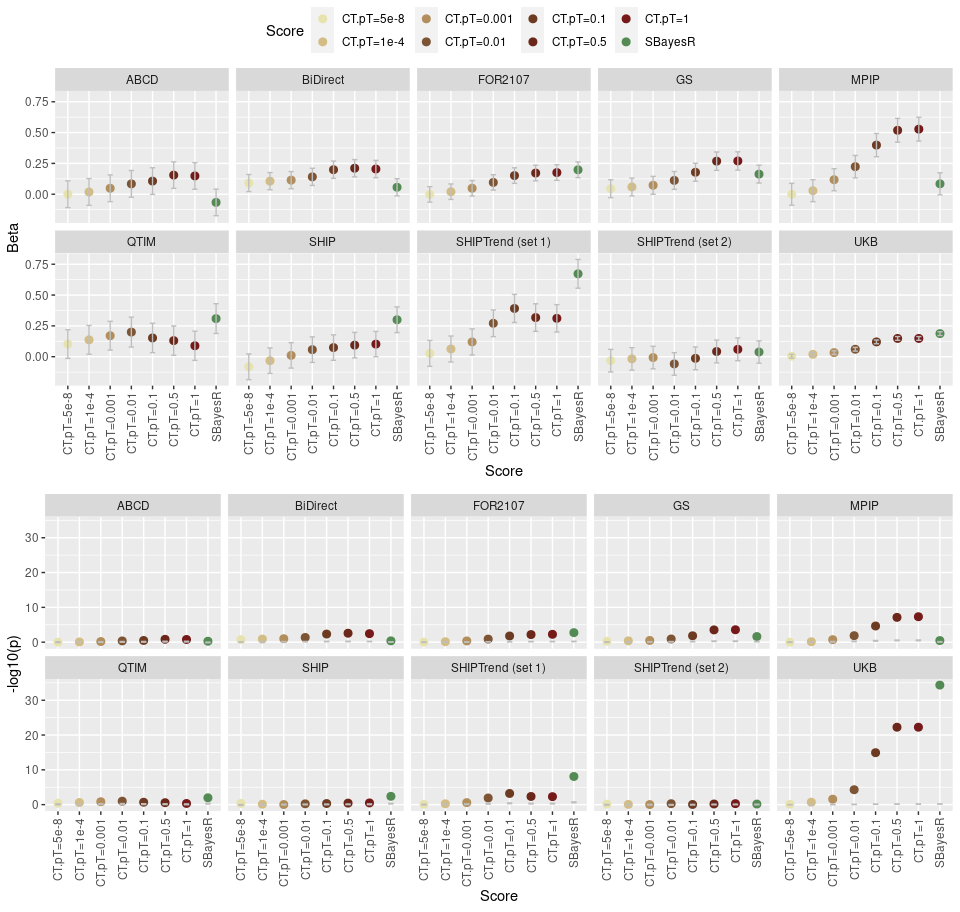


##### Supplementary Figure 4. Association between all MD PRS and global neuroimaging measures in adults (age ≥ 25 years) and youths (age < 25 years), respectively. X-axis represents individual PRS. CT=clumping and thresholding method, pT = p-value threshold used for creating PRS using clumping and threshold methods, and SBayesR = PRS created using the SBayesR-processed summary statistics. Y-axis represents β, i.e., standardised regression coefficient.


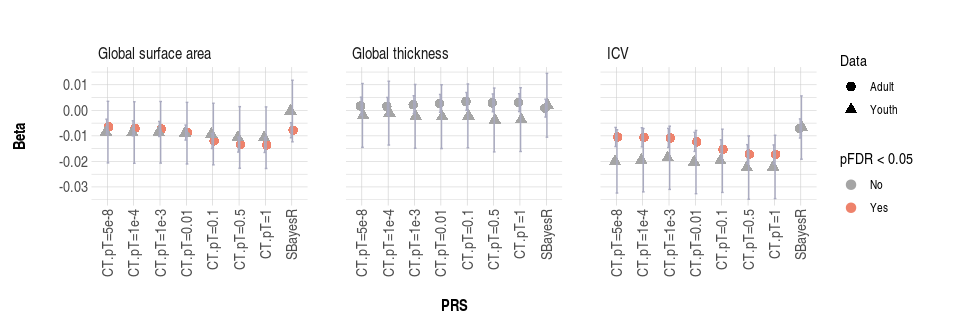


Supplementary Figure 5. Association between PRS-CT at pT=1 and brain regional measures. (a). P-value plot for associations between MD PRS and all regional measures. X-axis represents the three categories of brain regional measures. Y-axis represents -log10-transformed p-values. Each dot represents the result for one brain regional measure. Red and grey dashed lines are the whole-brain level FDR- and Bonferroni-significance threshold per category, respectively. Dark blue dashed line is the all-measure level FDR-significance threshold. Those associations that are significant after FDR correction are highlighted as solid dots. Top five FDR-significant associations per category are annotated with labels of regions in the figure. No associations were found in cortical thickness and therefore only the Bonferroni-significance threshold is shown in the figure. (b). Regional results for cortical surface areas. Darker colours represent lower beta-values (standardised regression coefficients). Regions that did not survive whole-brain FDR correction were coloured in grey. (c). Regional results for subcortical volumes in coronal view. Regions that did not survive whole-brain FDR correction were coloured in grey.


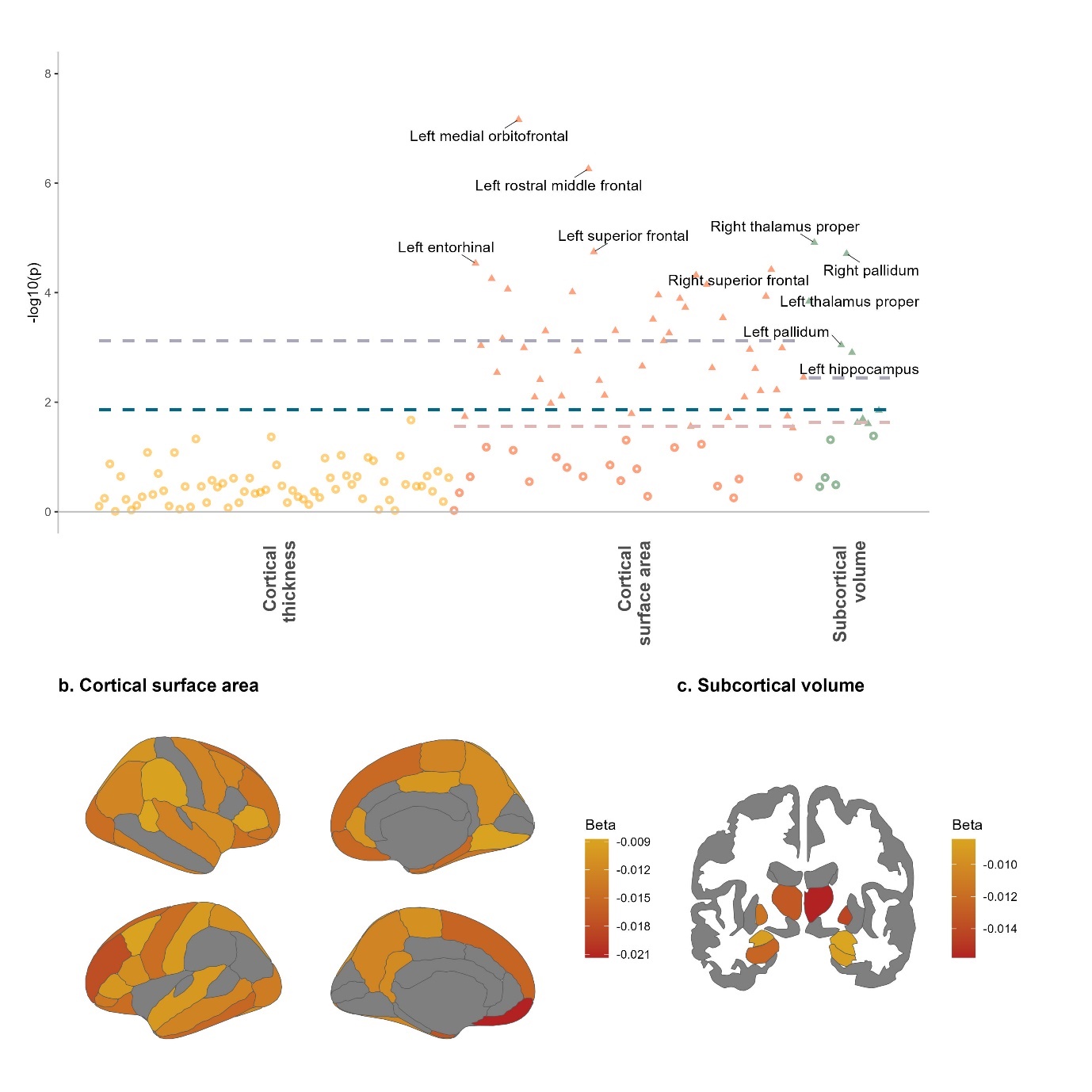


Supplementary Figure 6. Association between PRS-CT at pT=1 and brain regional measures with ICV as an additional covariate. (a). Regional results for cortical surface areas. Darker colours represent lower beta-values (standardised regression coefficients). (b). Regional results for subcortical volumes in coronal view.


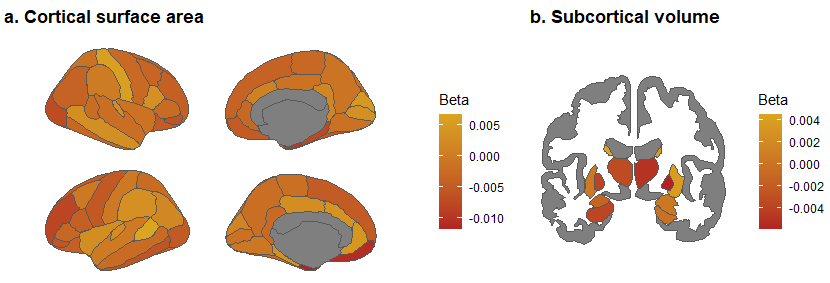


##### Supplementary Figure 7. Quality check of the potentially causal effect from left hippocampal volume to liability of MD.


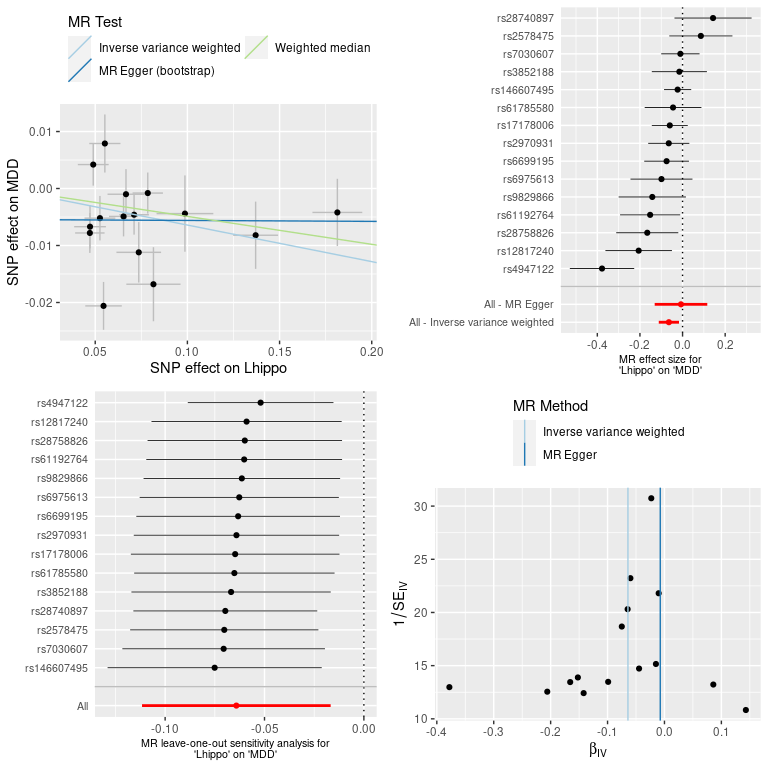


##### Supplementary Figure 8. LD matrix for the IVs used in the Mendelian randomisation analysis of causal effect from left hippocampal volume to liability of MD.


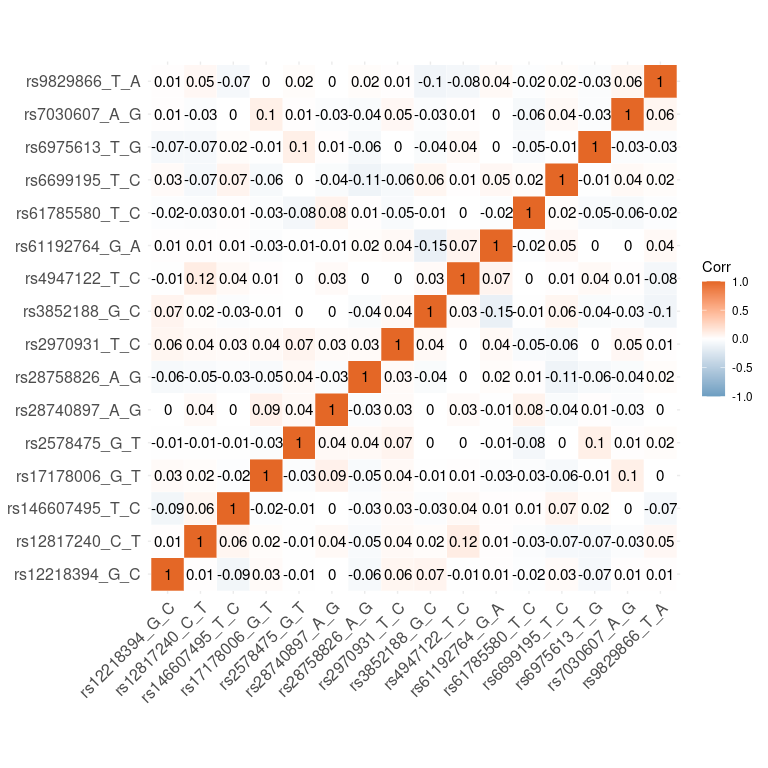


##### Supplementary Figure 9. LD matrix for the IVs used in the sensitivity Mendelian randomisation analysis of causal effect from left hippocampal volume to liability of MDD. Sensitivity analysis was conducted after removing rs4947122, rs28758826 and rs61192764 which showed medium level LD (r>0.15) with other IVs in the post-hoc quality check (Supplementary Figure 3).


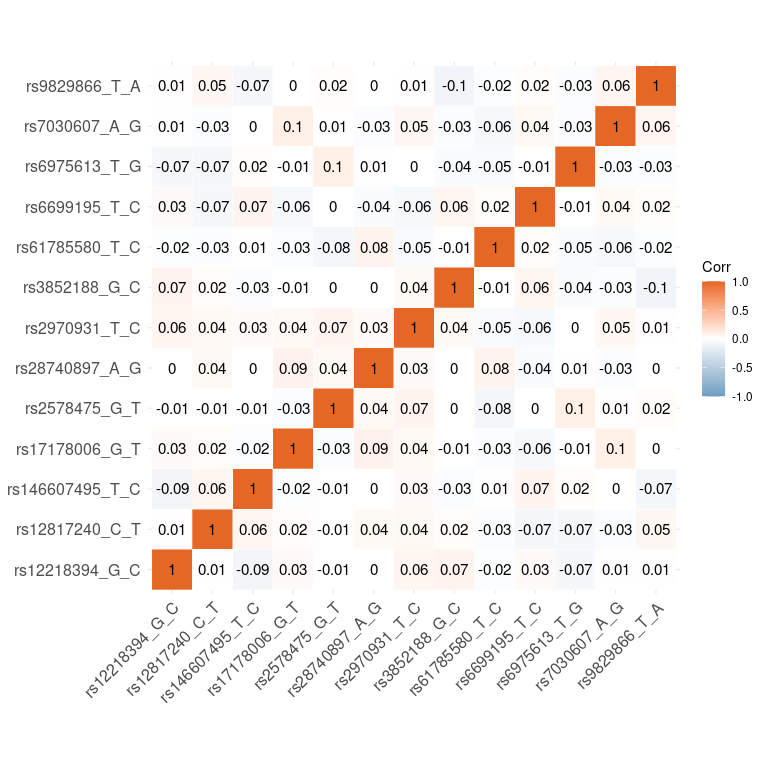


Supplementary Figure 10. Distributions of effect sizes of MD prediction using PRS-CT at p=1 and PRS-SBayesR. Log-transformed odds ratios were used as beta values.


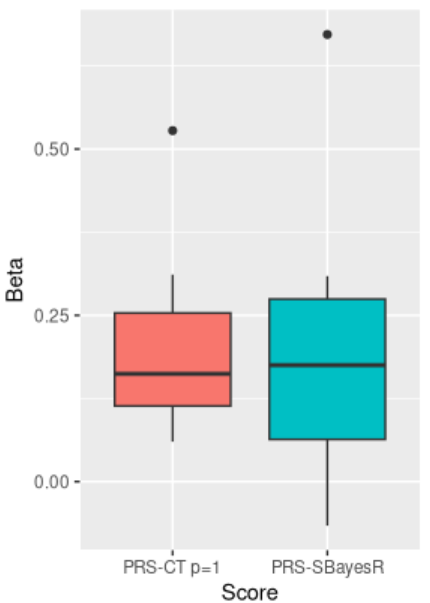


Supplementary Figure 11. Distributions of PRS-CT at p=1 (relatedness corrected). X-axis represents individual studies. Y-axis represents values of PRS-CT at p=1. GS_stradl = Generation Scotland.


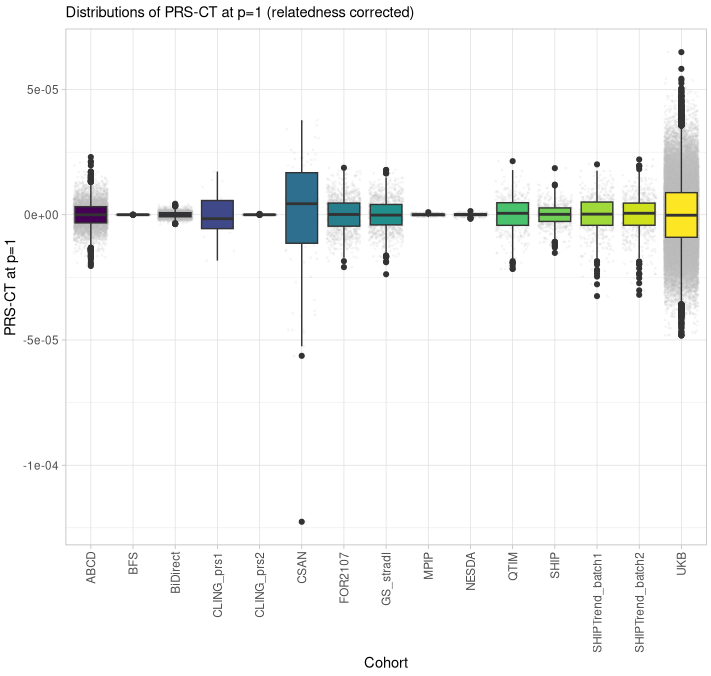


Supplementary Figure 12. Distributions of intracranial volumes (ICV). X-axis represents individual studies. Y-axis represents ICV. GS_stradl = Generation Scotland.


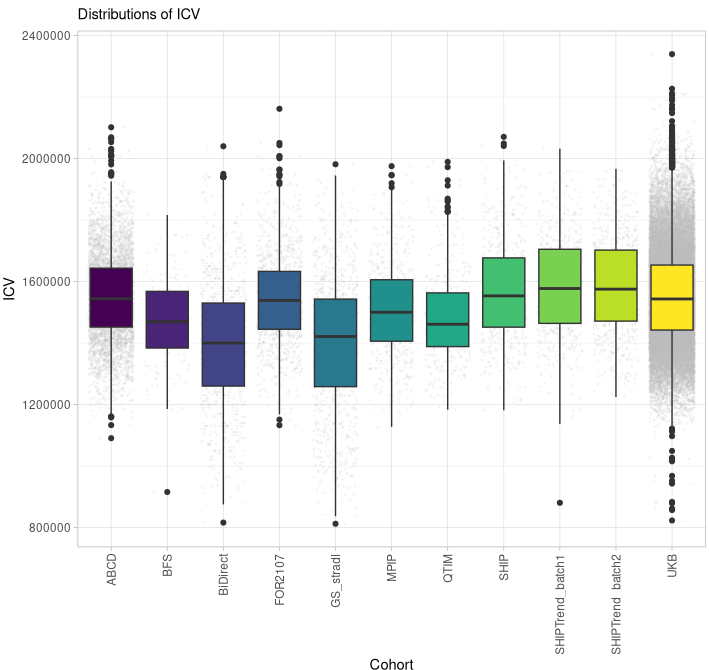


Supplementary Figure 13. Distributions of total cortical surface area. X-axis represents individual studies. Y-axis represents total cortical surface area. GS_stradl = Generation Scotland.


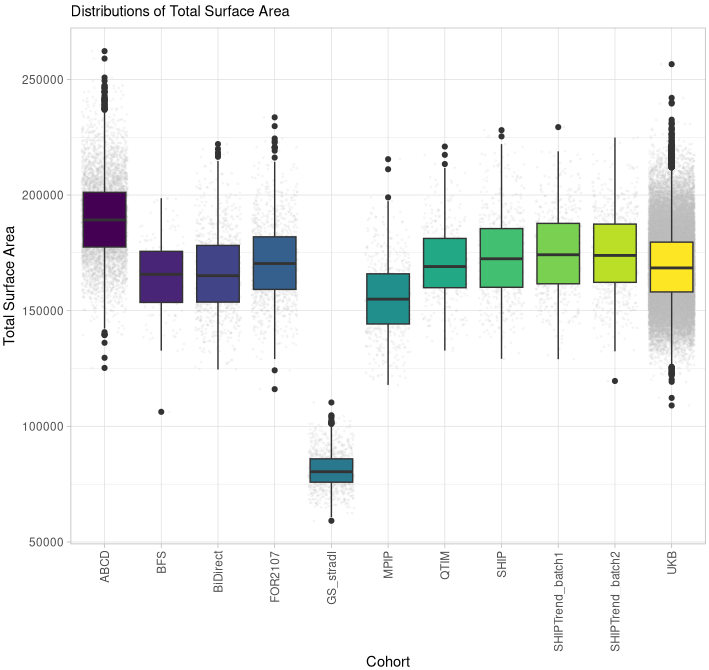


Supplementary Figure 14. Comparison of effect sizes between UKB and ENIGMA studies. X-axis represents global measures (total surface area, ICV and mean global thickness). Y-axis represents effect sizes (standardised regression coefficients) of associations with PRS-CT at p=1. Associations were null in the ENIGMA cohorts (p>0.24).


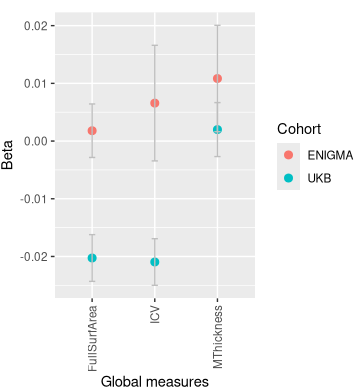


Supplementary Figure 15. Forest plot of associations between global measures and PRS-CT at p=1. X-axes represent standardised regression coefficients. Y-axes represent studies included in the mega analysis. GS_stradl = Generation Scotland.


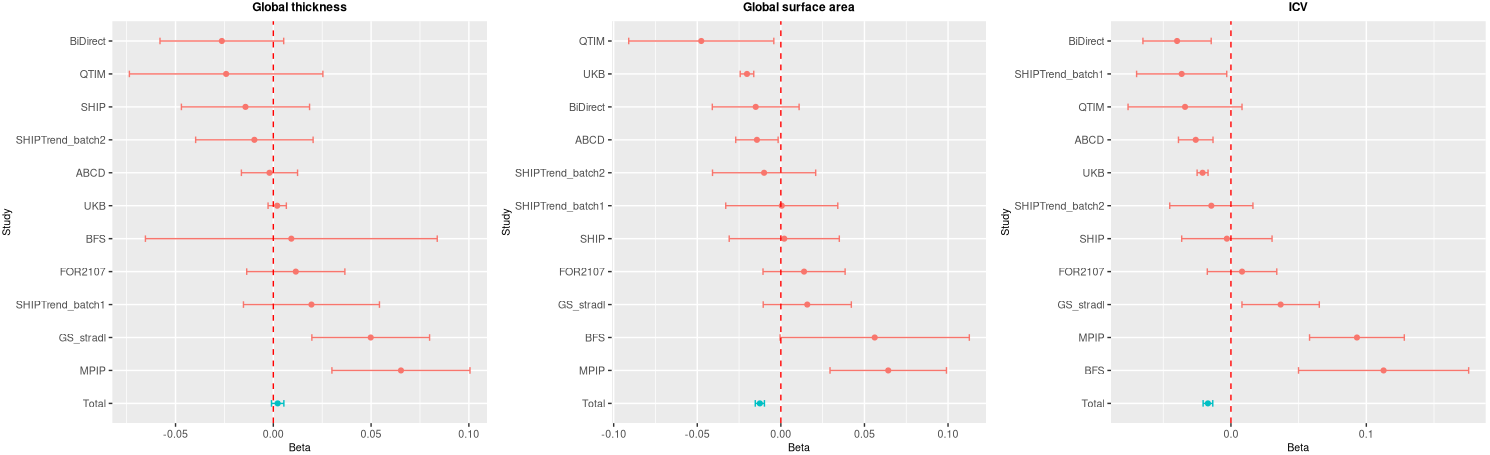


Supplementary Figure 16. Interaction effect of sex and PRS-CT at pT=1 in brain regional measures. X-axis represents the three categories of brain regional measures. Y-axis represents -log10-transformed p-values. Each dot represents the result for one brain regional measure. The grey dashed line is the whole-brain Bonferroni-significance threshold.


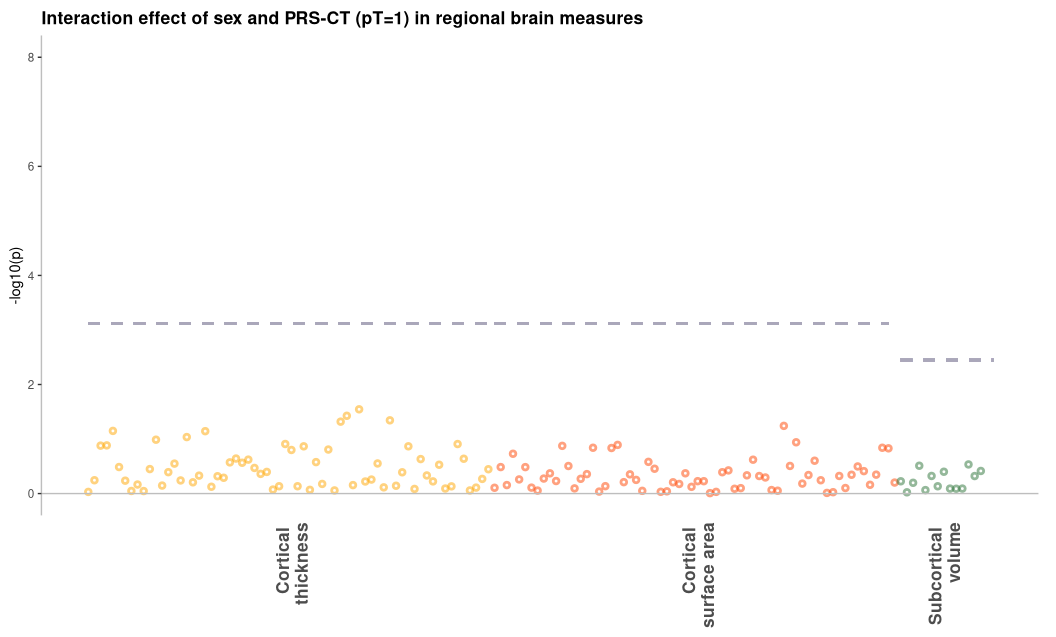


##### Supplementary Datasets

- Supplementary Data 1. Bidirectional Mendelian randomisation between brain structural measure and MDD.
